# Brain Regions Showing White Matter Loss in Huntington’s Disease Are Enriched for Synaptic and Metabolic Genes

**DOI:** 10.1016/j.biopsych.2017.10.019

**Published:** 2018-03-01

**Authors:** Peter McColgan, Sarah Gregory, Kiran K. Seunarine, Adeel Razi, Marina Papoutsi, Eileanoir Johnson, Alexandra Durr, Raymund A.C. Roos, Blair R. Leavitt, Peter Holmans, Rachael I. Scahill, Chris A. Clark, Geraint Rees, Sarah J. Tabrizi, Track-On HD Investigators, A. Coleman, J. Decolongon, M. Fan, T. Petkau, C. Jauffret, D. Justo, S. Lehericy, K. Nigaud, R. Valabrègue, A. Schoonderbeek, E.P. ‘t Hart, D. J. Hensman Moss, R. Ghosh, H. Crawford, M. Papoutsi, C. Berna, D. Mahaleskshmi, R. Reilmann, N. Weber, I. Labuschagne, J. Stout, B. Landwehrmeyer, M. Orth, I. Mayer, H. Johnson, D. Crawfurd

**Affiliations:** j University of British Columbia, Vancouver, Canada; k ICM and APHP, Pitié-Salpêtrière University Hospital, Paris, France; l Leiden University Medical Centre, Leiden, the Netherlands; m University College London, London, United Kingdom; n George Huntington Institute, Münster, Germany; o Monash University, Melbourne, Australia; p University of Ulm, Ulm, Germany; q University of Iowa, Iowa City, IA; r University of Manchester, Manchester, United Kingdom; a Huntington’s Disease Centre, Department of Neurodegenerative Disease, Queen Square, London, United Kingdom; b Wellcome Trust Centre for Neuroimaging, UCL Institute of Neurology, Queen Square, London, United Kingdom; c Developmental Imaging and Biophysics Section, UCL Institute of Child Health, Queen Square, London, United Kingdom; d National Hospital for Neurology and Neurosurgery, Queen Square, London, United Kingdom; e MRC Centre for Neuropsychiatric Genetics and Genomics, School of Medicine, Cardiff University, Cardiff, United Kingdom; f Department of Electronic Engineering, NED University of Engineering and Technology, Karachi, Pakistan; g APHP Department of Genetics, University Hospital Pitié-Salpêtrière; and ICM (Brain and Spine Institute) INSERM U1127, CNRS UMR7225, Sorbonne Universités – UPMC Paris VI UMR_S1127, Paris, France; h Department of Neurology, Leiden University Medical Centre, Leiden, the Netherlands; i Centre for Molecular Medicine and Therapeutics, Department of Medical Genetics, University of British Columbia, Vancouver, British Columbia, Canada

**Keywords:** Connectome, Genetics, Huntington's disease, Imaging, Transcription, White matter

## Abstract

**Background:**

The earliest white matter changes in Huntington’s disease are seen before disease onset in the premanifest stage around the striatum, within the corpus callosum, and in posterior white matter tracts. While experimental evidence suggests that these changes may be related to abnormal gene transcription, we lack an understanding of the biological processes driving this regional vulnerability.

**Methods:**

Here, we investigate the relationship between regional transcription in the healthy brain, using the Allen Institute for Brain Science transcriptome atlas, and regional white matter connectivity loss at three time points over 24 months in subjects with premanifest Huntington’s disease relative to control participants. The baseline cohort included 72 premanifest Huntington’s disease participants and 85 healthy control participants.

**Results:**

We show that loss of corticostriatal, interhemispheric, and intrahemispheric white matter connections at baseline and over 24 months in premanifest Huntington’s disease is associated with gene expression profiles enriched for synaptic genes and metabolic genes. Corticostriatal gene expression profiles are predominately associated with motor, parietal, and occipital regions, while interhemispheric expression profiles are associated with frontotemporal regions. We also show that genes with known abnormal transcription in human Huntington’s disease and animal models are overrepresented in synaptic gene expression profiles, but not in metabolic gene expression profiles.

**Conclusions:**

These findings suggest a dual mechanism of white matter vulnerability in Huntington’s disease, in which abnormal transcription of synaptic genes and metabolic disturbance not related to transcription may drive white matter loss.

Huntington’s disease (HD) is a progressive fatal neurodegenerative disease caused by a CAG repeat expansion in the *HTT* gene on chromosome 4. Individuals with more than 39 CAG repeats are certain to develop HD, allowing investigation of the premanifest stage (preHD) many years before symptom onset (1). While the caudate and putamen show the earliest gray matter changes (2), white matter (WM) changes are seen around the striatum, within the corpus callosum, and in the posterior WM tracts 2, 3, 4, 5. We have demonstrated a hierarchy of WM vulnerability where corticostriatal connections show greatest changes in preHD and control participants, followed by interhemispheric and intrahemispheric connections (6).

Voxel-based morphometry suggests 2, 7 that gray matter and WM abnormalities in the striatum occur in parallel in those furthest from disease onset, but more recent work (5) suggests that gray matter atrophy precedes WM atrophy in the striatum. However, as this was a cross-sectional study it is not yet possible to define a typical time lag. Thus, patterns of WM loss in preHD are well established, but the underlying pathological processes are unclear.

Mutant huntingtin protein causes cellular dysfunction and ultimately neuronal cell death through several processes 8, 9, including downstream effects on synaptic signaling (10), cellular metabolism (11), mitochondrial dysfunction (12), immune activation (13), and alterations in transcription (14). Furthermore, transcription levels of genes involved in these processes are atypical in human HD and animal models 14, 15. Decreased expression of synaptic proteins in cortical pyramidal neurons of HD mouse models are linked to abnormal corticostriatal connectivity (16), while changes in transcription levels of brain-derived neurotrophic factor, another protein involved in synaptic transmission, are associated with changes in corticocortical connectivity (17). Excitotoxic striatal lesion models of HD are consistent with these findings. Reduced brain-derived neurotrophic factor is seen in the rat striatum after quinolinic acid injection (18), and reduced brain-derived neurotrophic factor and nerve growth factor are seen after 3-nitropropionic acid treatment (19).

Some genes show a direct association with WM integrity. Loss of peroxisome proliferator–activated receptor gamma coactivator 1-alpha, involved in the transcriptional regulation of energy metabolism, results in striatal degeneration and corpus callosum WM abnormalities in HD mouse models (20). Reduced transcription levels of myelin-related genes are associated with WM abnormalities in HD mouse models (21).

Given the relationship between WM connectivity and gene transcription in HD, here we investigated how regional gene transcription profiles of the healthy human brain, obtained from the Allen Institute for Brain Science (AIBS) human transcriptome atlas (22), were associated with WM connectivity loss in preHD. Based on association between synaptic and metabolic genes and WM loss in HD 20, 21 we hypothesized that WM connectivity loss in preHD would be associated with regional transcription profiles enriched for synaptic and metabolic genes.

## Methods and Materials

### Overview

To test our hypothesis, WM connectivity loss was determined using diffusion-weighted imaging from a longitudinal cohort of preHD and control participants. Brains were parcellated into 70 cortical and 2 subcortical (caudate and putamen) regions of interest (ROIs) based on the Desikan FreeSurfer atlas (23). The caudate and putamen were chosen as these regions show the greatest changes in preHD (2). Whole-brain tractography was performed using these parcellations to construct WM brain networks. We have recently published a longitudinal analysis using this cohort (6).

**Figure 1:**
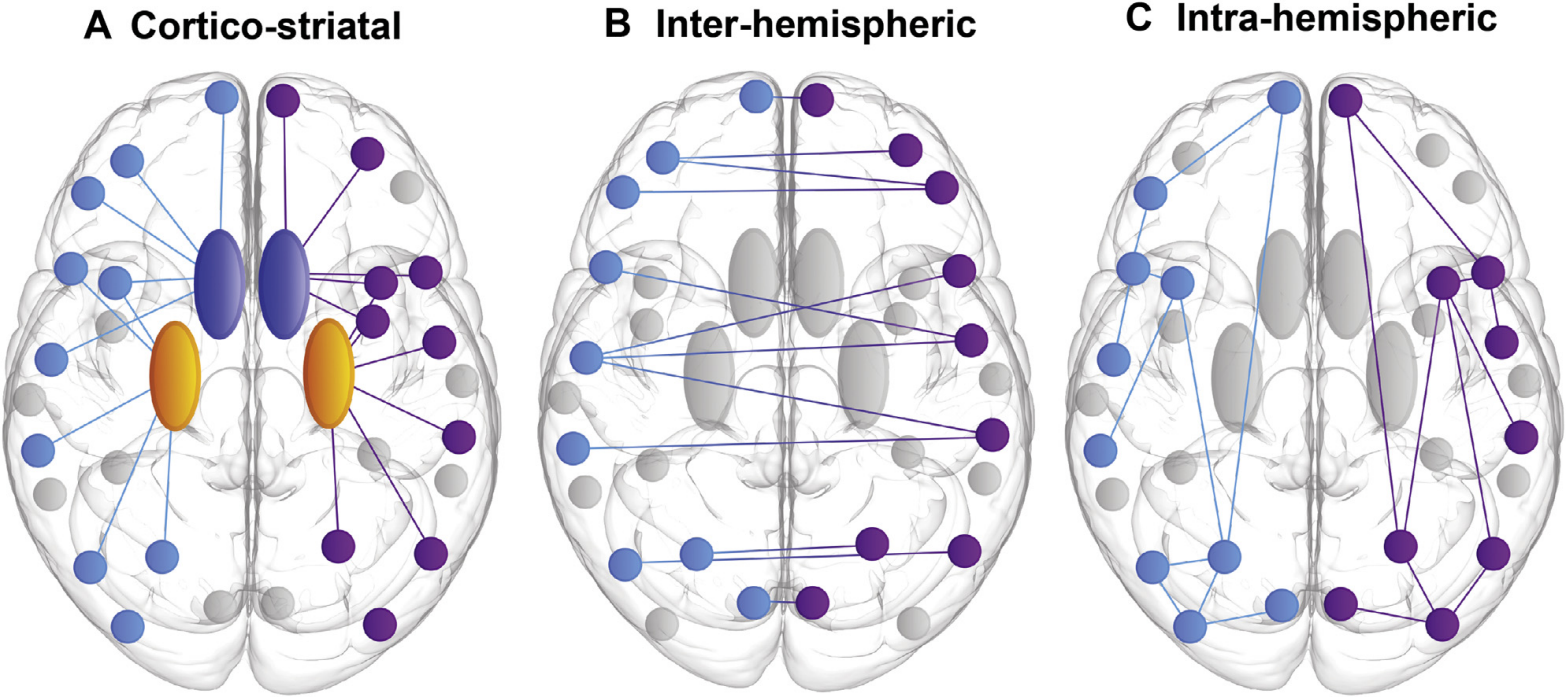
Schematic illustrating subgroups of regional white matter connectivity. **(A)** Corticostriatal: connections between cortex and striatum (caudate and putamen) for each cortical region of interest. **(B)** Interhemispheric: connections to the opposite hemisphere for each cortical region of interest. **(C)** Intrahemispheric: connections within the same hemisphere for each cortical region of interest. Light blue indicates the left hemisphere, purple indicates the right hemisphere, dark blue indicates the caudate, and yellow indicates the putamen.

For each set of connections associated with a cortical ROI, WM connectivity loss was defined as corticostriatal (connections between cortex and caudate/putamen), interhemispheric (corticocortical connections between hemispheres), or intrahemispheric (corticocortical connections within the same hemisphere) (see Figure 1). WM connectivity and rate of change in WM connectivity over 24 months were normalized for preHD relative to control participants for each participant. Connectivity measures were then transformed to give atrophy and rate of atrophy measures. The resulting atrophy score was used in the cross-sectional analysis, while the rate of atrophy score was used in the longitudinal analysis.

To compare regional WM loss in preHD with regional gene expression in the healthy brain, the 70 cortical ROIs (23) were matched to the closest AIBS ROI, and gene expression data were averaged across RNA probes corresponding to the same gene. ROIs with gene expression values >2 SD above the mean or range were excluded this resulted in the inclusion of 20,737 genes across 68 cortical ROIs.

Partial least squares (PLS) regression was used to investigate the relationship between regional gene expression and regional WM loss. PLS is a multivariate technique used when the number of predictor variables (i.e., regional gene expression) is much larger than the number of observations (i.e., regional WM loss). It has been used previously to investigate the relationship between gene expression and magnetic resonance imaging (MRI)–derived regional brain measures in healthy volunteers 24, 25. For our analysis the predictor variable comprised a gene × ROI matrix of 20,737 × 68, and the response variable comprised a WM loss × ROI matrix: 68 × 4 for the corticostriatal analysis (68 cortical ROIs × left and right caudate and putamen WM loss to each ROI region) and 68 × 1 for the inter- and intrahemispheric analyses (68 cortical ROIs × inter- and intrahemispheric WM loss for each ROI). PLS identified components or patterns of regional gene expression having maximum covariance with regional WM loss, such that the first few PLS components provide the greatest representation of the covariance. For each component, individual genes are assigned weights based on their contribution to the variance explained (24).

This analysis provided a weight for each gene indicating its contribution to WM connectivity loss for each component or pattern. Using this information, genes were ranked according to their PLS weight. Gene enrichment analysis was then performed to identify the biological functions of genes with the highest weights using gene ontology (GO) terms (26). Here, the significance of a GO term was determined based on the rank of genes associated with that term.

### Imaging Cohort

The cohort included preHD and control participants from the Track-On HD study (27), followed up at 3 time points over 24 months at four sites (London, United Kingdom; Leiden, the Netherlands; Paris, France; and Vancouver, British Columbia, Canada). Baseline participants included 72 preHD and 85 control participants. For the longitudinal analysis only preHD participants with diffusion data from all 3 time points were included (56 preHD and 65 control participants; see Supplemental Methods).

### MRI Acquisition

T1 and diffusion-weighted images were acquired on two different 3T MRI scanners (Philips Achieva [Philips Healthcare, Eindhoven, The Netherlands] at Leiden and Vancouver, and Siemens TIM Trio [Siemens Corp., Erlangen, Germany] at London and Paris). Diffusion-weighted images were acquired with 42 unique gradient directions (*b* = 1000 s/mm^2^; see Supplemental Methods).

### Diffusion Tractography

Whole-brain probabilistic tractography was performed using MRtrix Version 3.0 (28). The spherical-deconvolution informed filtering of tractograms 2 algorithm (29) was used to reduce biases. To demonstrate that our results were robust to varying methodologies, additional cross-sectional analyses used alternative connectome construction methodologies (see Supplemental Methods).

### Mapping Gene Expression Data to MRI Space

Gene expression microarray data were used from the AIBS atlas (22). Maybrain software (https://github.com/rittman/maybrain) matched centroids of MRI regions to the closest AIBS region. For the cross-sectional analyses a leave-one-out approach and three of six permutations of AIBS brain samples were also used to ensure that results were robust to different combinations of AIBS subjects (see Supplemental Methods).

### Statistical Analysis

PLS regression was used to investigate the association between gene transcriptome of the healthy brain and WM connectivity loss in preHD. Code used to perform this analysis was adapted from Whitaker *et al.* (25). Random permutations of the gene predictor variable were also investigated to ensure that results were not due to chance (see Supplemental Methods).

### GO Enrichment Analysis

We used the GO enrichment analysis and visualization tool (GOrilla) (http://cbl-gorilla.cs.technion.ac.il) (26) to identify GO terms that were significantly enriched in the target gene list.

### Overlap Between Gene Profiles and HD-Related Genes

To investigate similarities between gene profiles, we identified the significance of gene overlap between analyses using a hypergeometric distribution. GO enrichment analysis was also repeated with overlap genes removed to assess whether this affected the resulting GO terms. Overlap between genes in top GO terms and HD genes was also investigated.

### Enrichment for HD-Related Genes

We investigated whether genes showing abnormal transcription in human and animal models of HD were enriched greater than chance in the first PLS components of the corticostriatal, interhemispheric, and intrahemispheric analyses. HD gene lists were obtained from Langfelder *et al.* (30). Additionally we investigated whether HD-related genes were more strongly enriched in these gene lists than other biologically plausible gene sets, chosen at random. Gene sets for human supragranular genes, oligodendrocytes, and cell cycle metabolism were also investigated (see Supplemental Methods).

## Results

### Gene Expression Profiles of the Healthy Human Brain Explain the Variance of Regional WM Connectivity Loss in preHD

For the majority of analyses the first PLS component accounted for a large percentage of the variance in regional WM loss. We therefore focused on this component. Gene expression data explained 66% of the variance of regional WM connectivity loss in the corticostriatal cross-sectional analysis and 70% in the longitudinal analysis for the first component of the PLS and 11% and 6%, respectively, for the second component. For the interhemispheric analysis, gene expression explained 67% WM loss cross-sectionally and 17% longitudinally for the first component and 9% and 60%, respectively, for the second component. For the intrahemispheric analysis, gene expression explained 24% cross-sectionally and 65% longitudinally for the first component and 47% and 11%, respectively, for the second component. See Supplemental File 1 for the first component PLS gene weights for these analyses.

For each analysis the first components of the PLS were explored. The second components were also explored if they accounted for a large proportion of the variance. Variances explained by the first component ranged between 45% and 69% for random permutations of the gene predictor matrix; however, gene and ROI weights were very different from the original analysis.

### Expression Profiles Associated With Cross-Sectional Variation in WM Connections in preHD Relative to Control Participants

**Figure 2:**
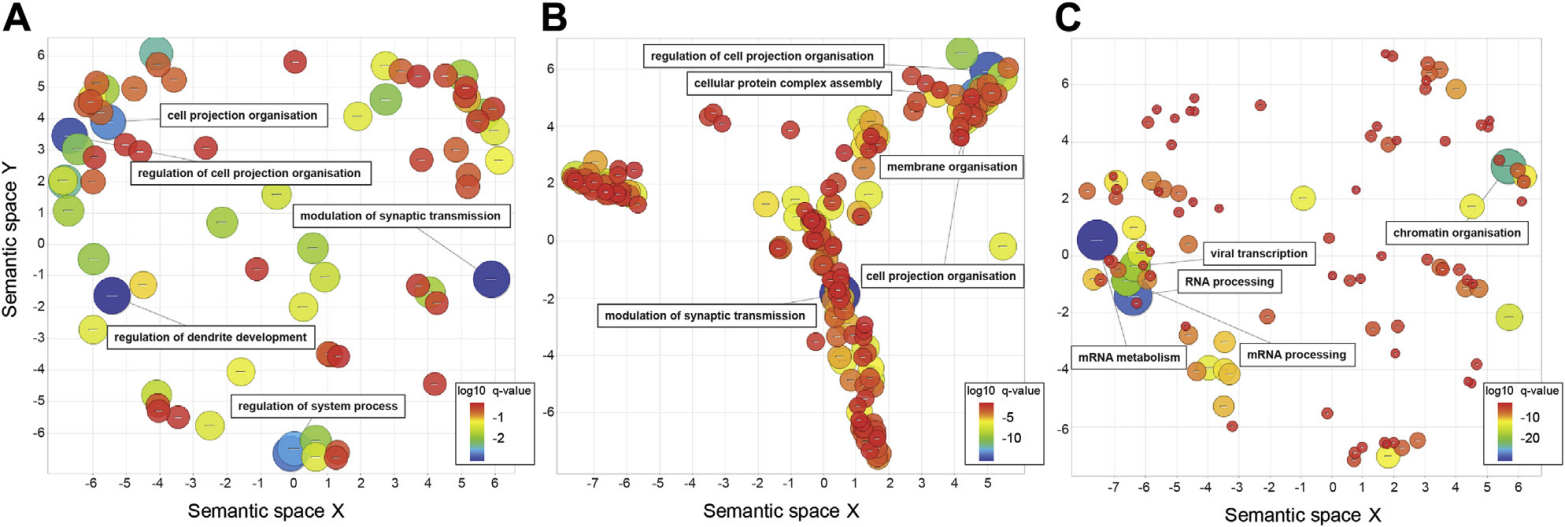
Significant gene ontology (GO) terms for biological processes associated with the first component of the partial least squares analysis are plotted in semantic space, where similar terms are clustered together. **(A)** Corticostriatal cross-sectional analysis semantic similarity scatter plot. **(B)** Interhemispheric cross-sectional analysis semantic similarity scatter plot. **(C)** Intrahemispheric cross-sectional analysis semantic similarity scatter plot. In all plots, the top five most significant GO terms are labeled for each analysis. Redundant GO terms and those associated with greater than 1000 genes have been excluded. Markers are scaled based on the log10 *q* value for the significance of each GO term. Large blue circles are highly significant, while red circles are less significant (see color bar). mRNA, messenger RNA.

Similar significant GO terms were seen for the corticostriatal and interhemispheric analyses including modulation of chemical synaptic transmission, regulation of cell projection organization, and cell projection organization. We refer to these as a synaptic profile. For the intrahemispheric analysis the most significant GO terms included messenger RNA (mRNA) metabolic process, RNA processing, and chromatin organization (see Table 1 and Figure 2), which we refer to as a metabolic/chromatin profile. For the intrahemispheric analysis the second component of the PLS was significantly associated with GO terms involved in myelination and lipid metabolism. See Supplemental File 2 for all significant GO terms for each analysis.

**Table 1:** Corticostriatal, Interhemispheric, and Intrahemispheric Cross-Sectional Analyses

GO Term	Description	*p* Value	FDR *q* Value	Enrichment^a^	B^b^	*n*^c^	*b*^d^
PLS1 Corticostriatal Cross-Sectional							
GO:0050773	Regulation of dendrite development	8.05E-07	3.03E-03	2.18	124	3150	48
GO:0050804	Modulation of chemical synaptic transmission	1.06E-06	3.19E-03	1.4	297	6419	151
GO:0031344	Regulation of cell projection organization	1.88E-06	4.06E-03	1.29	549	6375	255
GO:0044057	Regulation of system process	3.31E-06	4.98E-03	1.33	481	5795	209
GO:0030030	Cell projection organization	4.31E-06	5.41E-03	1.24	699	6498	319
PLS1 Interhemispheric Cross-Sectional							
GO:0050804	Modulation of chemical synaptic transmission	1.40E-14	3.51E-11	1.74	297	5246	153
GO:0031344	Regulation of cell projection organization	1.99E-13	2.73E-10	1.64	549	3924	199
GO:0043623	Cellular protein complex assembly	7.20E-13	8.34E-10	1.65	371	4892	169
GO:0061024	Membrane organization	1.51E-11	1.19E-08	1.45	820	4221	283
GO:0030030	Cell projection organization	6.38E-11	3.85E-08	1.47	699	4281	248
PLS1 Intrahemispheric Cross-Sectional							
GO:0016071	mRNA metabolic process	2.91E-33	1.12E-30	1.84	593	5085	313
GO:0006396	RNA processing	4.39E-30	1.58E-27	1.65	806	5357	402
GO:0006325	Chromatin organization	1.11E-25	3.73E-23	1.79	657	4364	289
GO:0006397	mRNA processing	4.33E-21	1.42E-18	1.78	402	5435	219
GO:0019083	Viral transcription	2.79E-20	8.77E-18	3.01	99	4044	68

Gene ontology (GO) terms for biological processes associated with top-ranking genes from the first component of the partial least squares (PLS) analysis. The top five most significant GO terms are displayed for each analysis. Full tables can be found in Supplementary File 2. Redundant GO terms and those associated with >1000 genes have been excluded.

FDR, false discovery rate; mRNA, messenger RNA.

a Enrichment = (*b*/*n*) / (B/total number of genes). See Eden *et al*. (26) for further details.

b Total number of genes associated with a specific GO term.

c Number of genes in the target set.

d Number of genes in the intersection.

The leave-one-out analyses showed that modulation of chemical synaptic signaling and cell projection organization were the most significant GO terms for corticostriatal and interhemispheric connections for nearly all permutations. For intrahemispheric connections, the GO terms mRNA metabolic process, RNA processing, and chromatin organization were among the most significant for all permutations. Similarly the addition of Gaussian noise also revealed consistent results (see Supplemental File 3). The 3 out of 6 permutation analyses revealed similar findings across many of the 8 permutations (see Supplemental File 4).

The use of fractional anisotropy (FA) weighting and the thresholded scale 60 easy Lausanne atlas resulted in a change from synaptic to metabolic/chromatin profiles for the corticostriatal and interhemispheric connections. For intrahemispheric connections FA weighting revealed a consistent metabolic/chromatin profile. For the thresholded scale 60 Lausanne atlas, intrahemispheric connections showed a synaptic profile. There was no change in profiles across consensus thresholds of 75% and 50%. Cross-sectional analyses using random permutations of genes revealed very different GO terms at minimal levels of significance, suggesting that our results are not due to chance (see Supplemental File 5).

### Expression Profiles Associated With Longitudinal Change in WM Connections in preHD Relative to Control Participants

For both corticostriatal and interhemispheric analyses, longitudinal change in WM was associated with GO terms involving metabolism or chromatin organization (see Supplemental Table S1 and Supplemental Figures S1 and S2). For intrahemispheric analysis, longitudinal change was associated with GO terns involved in mitochondrial function, metabolism, and synaptic transmission (see Supplemental Table S1 and Supplemental Figure S3). The second component of the PLS for the interhemispheric analysis was significantly associated with a range of GO terms including immune function, development, and protein folding (see Supplemental File 2). In summary, these results suggest that regional gene expression profiles associated with loss of WM connectivity in preHD are involved in synaptic, metabolic, and chromatin-related biological processes.

### Overlap Between Synaptic and Metabolic Gene Profiles and HD-Related Genes

A significant overlap of 346 genes (*p* < .001) was found between the top genes in the corticostriatal analysis and intrahemispheric analyses. These were then compared with the striatum genes showing transcriptional abnormalities in HD humans and animal models. This revealed eight genes in common, encoding proteins involved in cell cycle (*CEP135*), axon development (*NEK1*), and G protein coupling (*ADORA2A*; see Supplemental File 6). GO enrichment analysis with overlap genes removed did not change the most significant GO terms. The GO terms modulation of chemical synaptic transmission and mRNA metabolic process showed overlap of seven genes. HD-related genes showed overlap of 44 genes with the GO terms modulation of chemical synaptic transmission and 7 genes with mRNA metabolic process. The overlaps were not greater than those expected by chance.

### Dissociation of Corticostriatal, Interhemispheric, and Intrahemispheric Gene Enrichment in the Cortex

**Figure 3:**
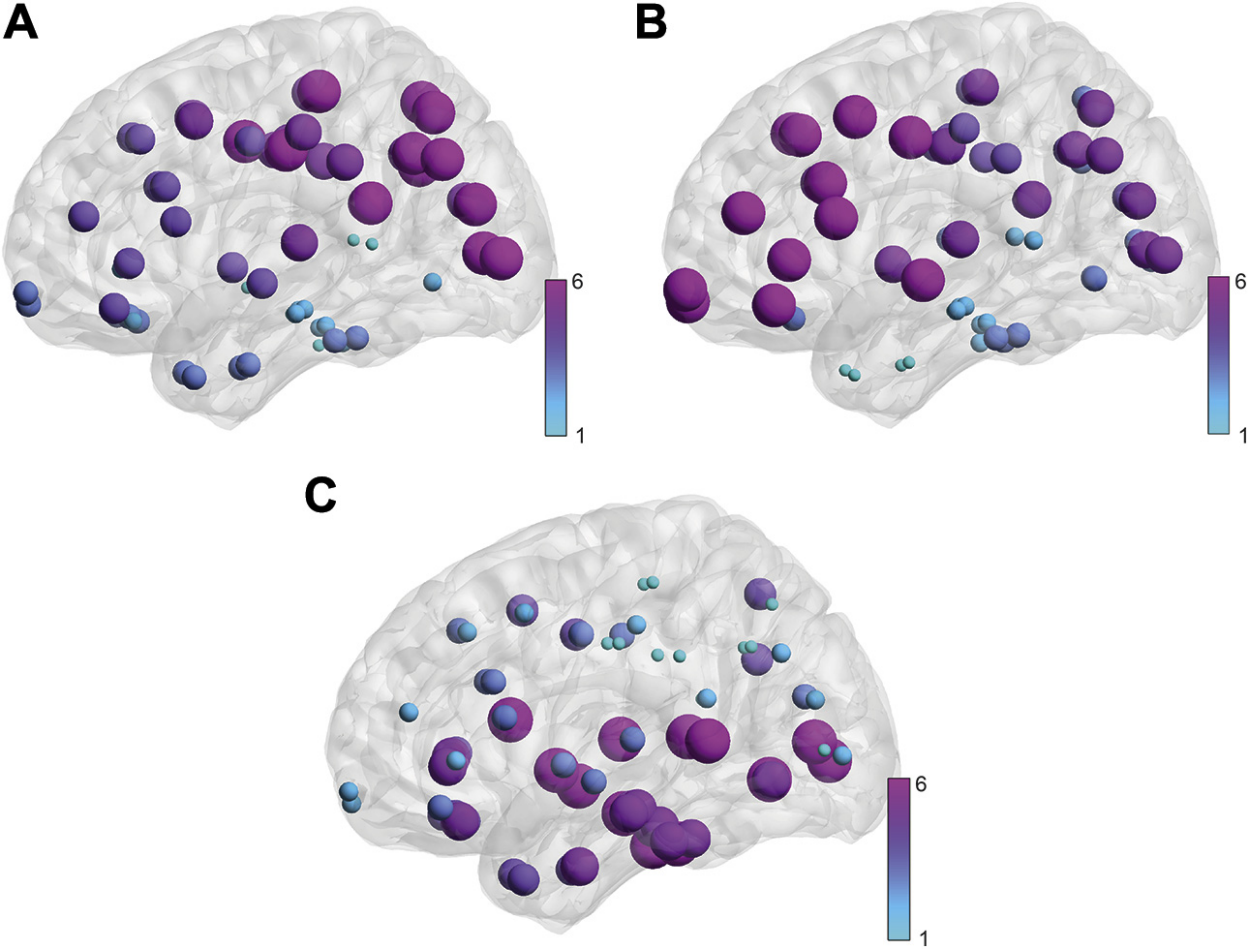
Region of interest weights for cross-sectional partial least squares regression analyses: **(A)** corticostriatal, **(B)** interhemispheric, and **(C)** intrahemispheric. Brain regions displayed on brain mesh. Size and color of region indicates size of region of interest weight (ranked from smallest [1] to largest [6]). See color map.

**Figure 4:**
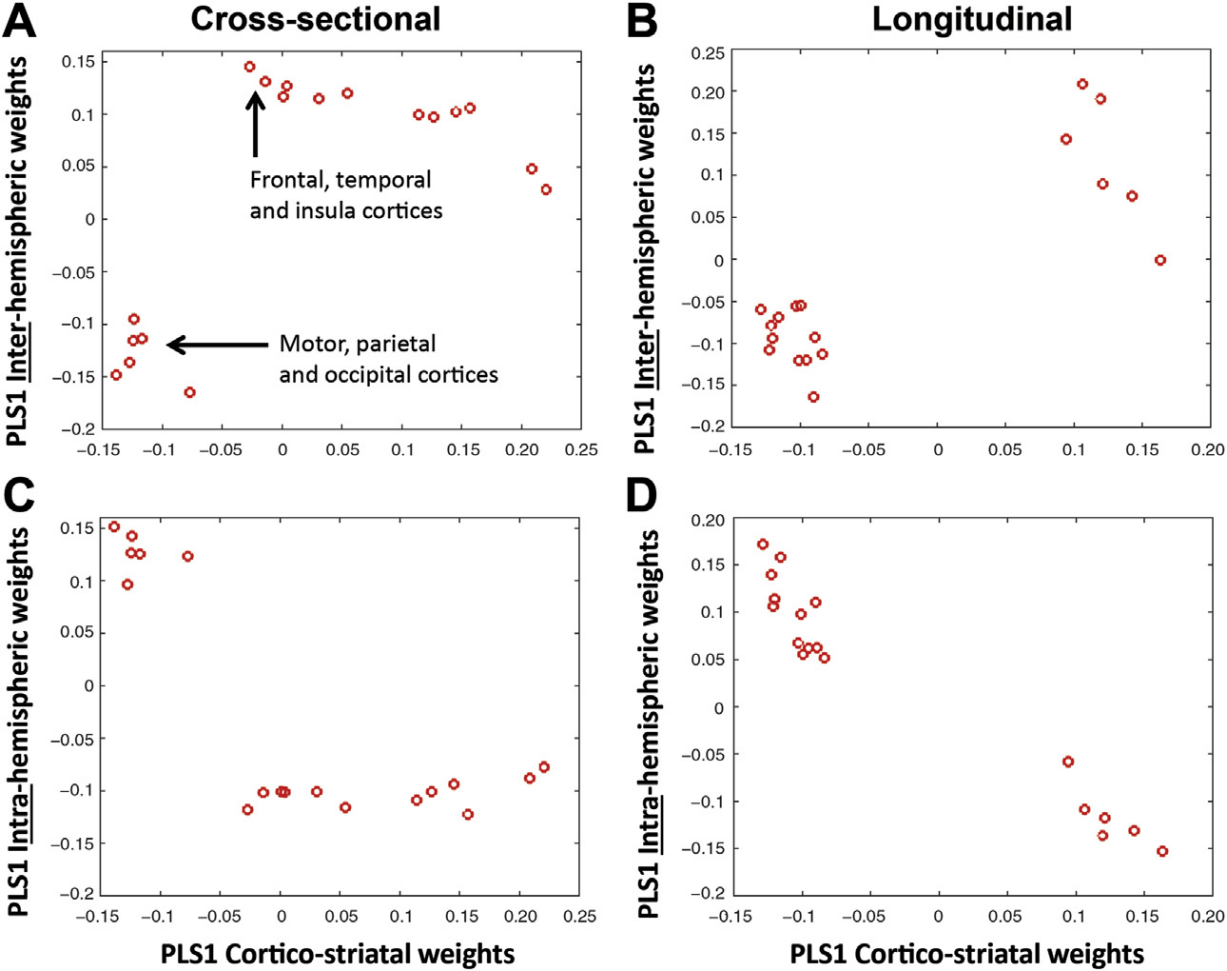
Dissociation of corticostriatal and inter- and intrahemispheric gene enrichment in the cortex. **(A)** Region of interest (ROI) weights for the first partial least squares (PLS) component of the cross-sectional analysis for interhemispheric vs. corticostriatal. **(B)** ROI weights for the first PLS component of the longitudinal analysis for interhemispheric vs. corticostriatal. **(C)** ROI weights for the first PLS component of the cross-sectional analysis for intrahemispheric vs. corticostriatal. **(D)** ROI weights for the first PLS component of the longitudinal analysis for intrahemispheric vs. corticostriatal. Each red circle represents a cortical ROI. PLS1, first partial least squares component.

The next step in our analysis was to explore the spatial pattern of each gene expression profile in the brain. To determine what brain regions were enriched with each gene expression profile, we analyzed PLS ROI weights from each analysis where higher weights related to greater gene profile enrichment (see Supplemental File 7 for ROI weights for each analysis). Cortical regions with the highest weights in the corticostriatal analysis (cross-sectional) were predominantly in motor, parietal, and occipital cortices. Conversely, cortical regions with the highest weights in the interhemispheric analysis (cross-sectional) were predominantly in frontal, temporal, and insular cortices. Cortical regions with the highest weights in the intrahemispheric analysis (cross-sectional) included frontal, temporal, and occipital regions (see Supplemental Table S2 and Figure 3). Plotting corticostriatal ROI weights against both interhemispheric and intrahemispheric ROI weights revealed dissociation in terms of regions involved, where regions enriched in the corticostriatal analysis were distinctly different from those enriched in the interhemispheric and intrahemispheric analyses (see Figure 4). Cross-sectional analyses using random permutations of ROIs revealed very different distribution of ROI weights, suggesting that our results are not due to chance (see Supplemental Figure S5 and Supplemental File 7).

### Enrichment of Genes Showing Abnormal Transcription in HD Is Seen in the Corticostriatal and Interhemispheric Gene Expression Profiles

**Figure 5:**
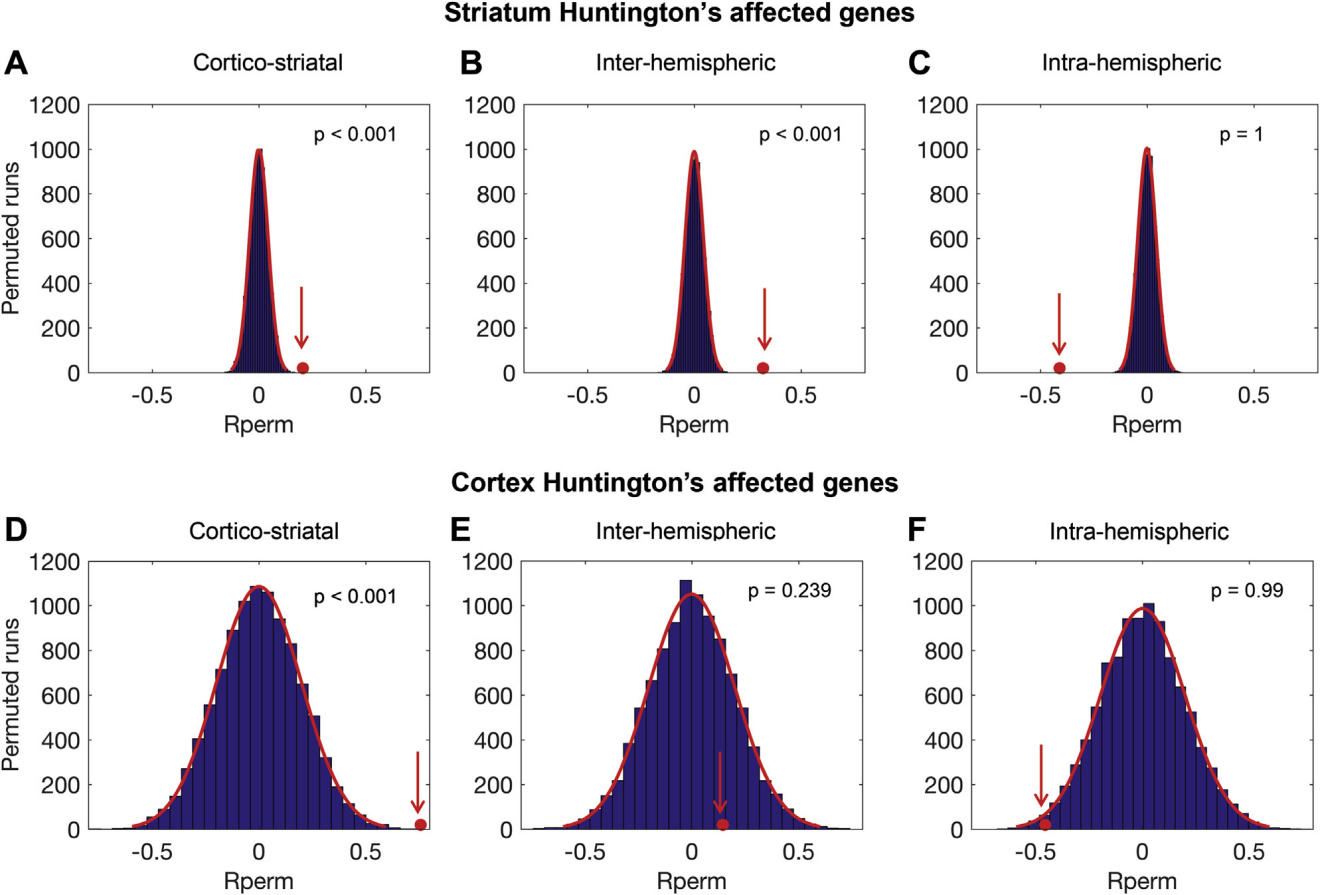
Enrichment of genes showing abnormal transcription in Huntington’s disease (HD) in the first partial least squares (PLS) component for the cross-sectional analyses. **(A)** Corticostriatal analysis, HD striatum genes. **(B)** Interhemispheric analysis, HD striatum genes. **(C)** Intrahemispheric analysis, HD striatum genes. **(D)** Corticostriatal analysis, HD cortex genes. **(E)** Interhemispheric analysis, HD cortex genes. **(F)** Intrahemispheric analysis, HD cortex genes. The red circle illustrates the mean weight (on the x axis) for the gene list of interest in the first PLS component. The y axis represents the number of permutations of random genes from the first PLS component. Gene lists overexpressed in the first PLS component have a mean greater than that of the random permutations (red circle to the right of the permutation distribution). Rperm, random permutation mean.

Our next step was to assess whether genes that show abnormal transcription in HD, both in the cortex and in the striatum, may be associated with WM loss. The corticostriatal gene list was significantly enriched for abnormal HD genes in the striatum (*p <* .001) and in the cortex (*p <* .001). The interhemispheric gene list was significantly enriched for genes in the striatum (*p <* .001), but not in the cortex. No significant enrichment was seen for the intrahemispheric gene list (see Figure 5). To ensure that the significance difference for the striatum gene list was not related to the size of the gene data set we repeated the analysis using the top 25 most significant genes based on the *q* value from Hodges *et al*. (31). Results were consistent with the 515 gene list showing significant enrichment for HD genes in the striatum for the corticostriatal (*p =* .019) and interhemispheric (*p =* .004) analyses (see Supplementary Figure S4). Enrichment compared against biologically plausible gene sets revealed similar results, for both 515 and 25 striatum gene lists, with significant enrichment for corticostriatal (*p <* .001) and interhemispheric analyses (*p <* .001) but not for the intrahemispheric analysis. This suggests that abnormal transcription in HD may be associated with corticostriatal and interhemispheric WM connectivity loss.

To further investigate the relationship between changes in gene expression in HD relative to control participants and corticostriatal WM loss, we performed correlations between the log2 fold change in the Hodges *et al.* (31), Durrenberger *et al.* (32), and Langfelder *et al.* (30) studies for the 515 striatum gene set and the PLS weights from the cross-sectional corticostriatal analysis. This revealed negative correlations between PLS weights and log2 fold change (Hodges *et al.* [ρ = −.23, *p* = 1.1 × 10^−7^], Durrenberger *et al.* [ρ = −.23, *p* = 8.4 × 10^−8^], Langfelder *et al.* [ρ = −0.19, *p* = 1.6 × 10^−5^]; see Supplemental Figure S6). This suggests that genes associated with corticostriatal WM loss in preHD are also those that show reduced levels of transcription in human HD and animal models.

### Enrichment for Other Gene Lists

We also investigated enrichment for genes associated with the human supragranular cortex, oligodendrocytes, and cell cycle metabolism. The corticostriatal and interhemispheric gene lists were significantly enriched for human supragranular cortex genes (corticostriatal: *p =* .002, interhemispheric: *p =* .006) and oligodendrocyte genes (corticostriatal: *p <* .001, interhemispheric: *p <* .001), but not for cell cycle metabolism genes. Conversely, the intrahemispheric gene list was significantly enriched for cell cycle metabolism genes (*p <* .001), but not for human supragranular or oligodendrocyte genes. This suggests a relationship between corticostriatal WM loss and abnormal transcription in oligodendrocytes. Additionally, abnormal transcription in cortical supragranular genes, which are implicated in long-range connectivity (33), may be linked to connectivity corticostriatal and interhemispheric WM loss.

## Discussion

In this study, we found that regional variance in WM loss in preHD is differentially associated with the pattern of expression of genes involved in synaptic, metabolic, and chromatin-related processes in the healthy human brain. Corticostriatal and interhemispheric WM loss is associated with synaptic genes, whereas intrahemispheric WM loss is associated with metabolic and chromatin-related genes. There is also a distinction between gene enrichment in cortical regions, where enrichment associated with corticostriatal connections is seen in more posterior regions such as motor, occipital, and parietal cortices, and gene enrichment associated with interhemispheric connections, which is seen in frontal, temporal, and insular cortices. We reveal that genes showing abnormal transcription in HD humans and animal models are overexpressed in the ranked gene list associated with corticostriatal and interhemispheric WM loss but not with intrahemispheric WM connection loss.

We focus on synaptic, metabolic, and chromatin-related genes to simplify interpretation of our results. However, specific GO terms such as DNA metabolism may relate to DNA repair (34). DNA repair genes, such as *MSH3*, have been linked to CAG instability (35), age of onset (36), and disease progression (34). The GO term mRNA metabolism may relate to splicing of mRNA, which has also been implicated in HD pathogenesis. Aberrant splicing of the mutant *HTT* gene leads to the generation of the pathogenic exon 1 huntingtin protein (37). We note that further work would be needed to link these specific gene sets directly to WM loss in HD.

Several studies have analyzed gene expression profiles in both human HD and animal models. Gene expression measured in postmortem brain samples from HD patients was most affected in the caudate, followed by the motor cortex, while no abnormalities were detected in the prefrontal association cortex (31). The GO term showing greatest significance for both the caudate and motor cortex was synaptic transmission. Furthermore, significance for the GO terms metabolism and glucose metabolism were seen in the cortex, but not in the caudate. These findings agree with the associations between synaptic genes and corticostriatal WM connection loss and metabolic genes and intrahemispheric WM connection loss that we demonstrate here.

In our previous longitudinal study, WM loss was greatest in corticostriatal and interhemispheric connections in preHD relative to control subjects. No group differences were seen in intrahemispheric connections (6). The analysis presented here is based on regional atrophy of connection subtype. Therefore, corticostriatal and interhemispheric regional atrophy is likely to be greater than intrahemispheric regional atrophy. Furthermore, corticostriatal and interhemispheric connections have greater topographical lengths than intrahemispheric connections (6). Therefore, these similarities between corticostriatal and interhemispheric connections may account for the similarity between gene profiles.

Changes from synaptic to metabolic profiles in cross-sectional versus longitudinal, streamline volume versus FA weighting, and Desikan versus scale 60 easy Lausanne atlas were seen for corticostriatal and interhemispheric connections. We investigated this further, showing that common genes highly ranked in both profiles. One explanation for this may be that atrophy scores cross-sectionally will be higher than longitudinal rate of atrophy scores. Similarly, atrophy scores in the Desikan 68-region atlas are likely to be larger than in the more finely parcellated easy Lausanne scale 60 (110-region) atlas. With respect to FA weighting, this metric is difficult to interpret in crossing fiber regions, which make up an estimated 60% to 90% of the human brain (38).

The GO categories identified contain large numbers of genes. We therefore balance this data-driven approach by investigating whether gene profiles associated with regional WM loss in preHD are enriched for genes known to show abnormal transcription in both human HD and animal models. Similar GO terms such as synaptic transmission and chromatin modification have been associated with functional brain networks in healthy participants 24, 39. This likely represents the close relationship between the healthy brain network and the perturbation of that network in neurodegeneration (40).

We acknowledge the limitations of diffusion tractography. To address these we used both constrained spherical deconvolution tractography, which deals more effectively with crossing fibers than the diffusion tensor or multitensor methods (28), and the spherical-deconvolution informed filtering of tractograms 2, which has higher reproducibility and is more representative of the underlying biology of WM connections than conventional methods (41). Constrained spherical deconvolution tractography performs well at the acquisition protocol specifications used in this study (*b* =1000) 42, 43. At *b* = 1000, a minimum number of 28 gradient directions is required (44). Therefore, the angular coverage achieved using constrained spherical deconvolution tractography at *b* = 1000 is more than sufficient, with 42 directions.

The use of gene expression data from the healthy human brain to explain WM loss in preHD is limited to the extent that transcription in preHD may be different than that seen in healthy brains. However, studies from postmortem manifest HD brains show that the transcription in the striatum is most affected, with limited abnormalities in the cortex (31). Indeed, the transcription of only 25 genes in the cortex is abnormal in both human and animal studies, compared with 515 in the striatum (30). Therefore, we mitigated for the likely transcription abnormalities in preHD by using only cortical gene expression data from the AIBS transcriptome atlas (45).

We mapped the anatomical location of ROIs to corresponding regions in the AIBS atlas. However, the resolution of these atlases are different, and thus we acknowledge that the correspondence may not be exact and may be a limitation of our methodology. There are other human brain transcriptome atlas such as Braineac (46) and the Human Brain Transcriptome project (47); however, these atlases offer low resolution compared with the AIBS atlas, in which only a small number of cortical regions have been sampled, so the analysis carried out in this study could not be reproduced using Braineac or the Human Brain Transcriptome project atlas.

The utility of using information from the healthy human brain to inform us about the patterns and mechanisms of neurodegeneration has been demonstrated many times in neuroimaging. Functional connectivity and WM networks from healthy participants can predict atrophy in Alzheimer’s disease, corticobasal syndrome, frontotemporal dementia, and Parkinson’s disease 41, 48, 49, 50. More recently, transcriptome data from the healthy brains of the AIBS atlas has been used to investigate the association between the expression of schizophrenia risk genes and WM disconnectivity (51). The regional expression of the tau gene *MAPT* from the AIBS atlas has also been linked to the selective vulnerability of highly connected brain regions in Parkinson’s disease and progressive supranuclear palsy (52).

### Conclusions

We show that corticostriatal and interhemispheric WM connection loss is associated with the expression of synaptic genes in preHD, while intrahemispheric WM loss is associated with metabolic genes. Genes showing abnormal transcription in HD are associated with the synaptic profiles, but not with metabolic gene profiles. These findings have important implications for linking the earliest WM changes in preHD to the underlying pathological processes that may drive them.
