# Supplementary material for: Brain Regions Showing White Matter Loss in Huntington’s Disease Are Enriched for Synaptic and Metabolic Genes
Source: Biol Psychiatry. 2018 Mar 1;83(5):456–65. doi: 10.1016/j.biopsych.2017.10.019 (PMC5803509; doi:10.1016/j.biopsych.2017.10.019)
Supplement: Supplemental Material [file mmc1.pdf]

Brain Regions Showing White Matter Loss in Huntington's Disease Are Enriched for Synaptic and Metabolic Genes

Supplemental Information

Supplemental Methods

Imaging Cohort

Track-On is an extension of the Track-HD (1) study, but with only preHD and control participants carried over (early HD participants from Track-HD were excluded). Informed consent was obtained from each participant, and the study protocol was approved by the local ethics committees. Of the participants included, 31 preHD and 29 controls had participated previously in Track-HD (1). The preHD participants required a disease burden score (DBS) > 250 (2), on the basis of their medical records at the time of assessment. Controls were selected from the spouses or partners of preHD individuals or were gene-negative siblings, to ensure consistency of environments. For this study, we excluded participants who had manifest disease at baseline, were left handed or ambidextrous, or had poor quality diffusion-weighted imaging (DWI) data, as defined by visual quality control. Therefore only preHD participants were included who have not yet developed the motor manifestations of HD.

MRI Acquisition

Data were acquired on two different 3T MRI scanners (Philips Achieva at Leiden and Vancouver and Siemens TIM Trio at London and Paris), both using a 12-channel head coil. T1-weighted image volumes were acquired using a 3D MPRAGE acquisition sequence with the following imaging parameters: TR = 2200ms (Siemens)/ 7.7ms (Philips), TE=2.2ms (S)/3.5ms (P), FA=10◦ (S)/8◦(P), FOV= 28cm (S)/ 24cm (P), matrix size 256x256

(S)/224x224 (P), 208 (S)/164 (P) sagittal slices to cover the entire brain with a slice thickness of 1.0 mm with no gap.

Diffusion-weighted images were acquired with 42 unique gradient directions ($b = 1000$ sec/mm^2). Eight images with no diffusion weighting ($b = 0$ sec/mm^2) and one image with no diffusion weighting ($b = 0$ sec/mm^2) were acquired from the Siemens and Philips scanners respectively. For the Siemens scanners, TE = 88ms and TR = 13s; for the Phillips scanners, TE = 56ms and TR = 11s. Voxel size for the Siemens scanners was 2 x 2 x 2 mm and for the Phillips scanners 1.96 x 1.96 x 2. Seventy-five slices were collected for each diffusion-weighted and non-diffusion weighted volume. Scanning time was approximately 12 minutes for T1-weighted and 10 minutes for diffusion-weighted acquisitions.

MRI Data Analysis

Structural MRI Data

Cortical and sub-cortical regions of interest (ROIs) were generated by segmenting a T1-weighted image using FreeSurfer (3). These included 70 cortical regions and 4 sub-cortical regions (caudate and putamen bilaterally). We chose to focus on the caudate and putamen sub-cortical structures based on observations from our cross-sectional structural connectivity study (4) and from the earlier Track-HD studies (5, 6) that show the caudate and putamen are the sub-cortical structures most affected in preHD both in terms of grey matter volume and white matter connections While some studies have shown changes in the thalamus, globus pallidus and nucleus accumbens in preHD these tend to occur in preHD participants closer to disease onset (7, 8). Furthermore automatic segmentation of globus pallidus, nucleus accumbens and amygdala are not sufficiently reliable (9).

We choose the Desikan FreeSurfer atlas as this is based on 40 subjects across a range of ages encompassing 4 groups; young adults, middle aged adults, elderly adults and patients with Alzheimer's disease. By including subjects with age and neurodegenerative related atrophy this better accounts for inter-subject variability (3), particularly in the case of our cohort, which contains adults across a range of ages and those with preHD. We have used this atlas extensively in HD, for both cross-sectional and longitudinal connectome analyses (4, 10-12). Atlases with large numbers of ROIs demonstrate less reproducibility (13). While the AAL atlas is commonly used in graph theory studies this is derived from single subject who was young and healthy and is therefore not suitable for the cohort investigated here (14).

Data Pre-processing

For the diffusion data the b=0 image was used to generate a brain mask using FSL's brain extraction tool (15). Eddy current correction was used to align the diffusion-weighted volumes to the first b=0 image and the gradient directions updated to reflect the changes to the image orientations. Finally, diffusion tensor metrics were calculated and constrained spherical deconvolution (CSD) applied to the data as implemented in MRtrix (16). FreeSurfer Desikan atlas (3) ROIs were warped into diffusion space by mapping between the T1-weighted image and fractional anisotropy (FA) map using NiftyReg (17) and applying the resulting warp to each of the ROIs. A foreground mask was generated by combining FreeSurfer segmentations with the WM mask.

Diffusion Tensor Imaging Data

Diffusion Tractography

Whole brain probabilistic tractography was performed using the iFOD2 algorithm in MRtrix (16). Specifically, five million streamlines were randomly seeded throughout the WM, in all

foreground voxels where FA>0.2. Streamlines were terminated when they either reached the cortical or subcortical grey-matter mask or exited the foreground mask. The spherical deconvolution informed filtering of tractograms (SIFT2) algorithm (18) was used to reduce biases. The resulting set of streamlines was used to construct the structural brain network. To demonstrate our results were robust to varying methodologies additional cross-sectional analyses were completed using the addition of Gaussian noise to connectomes, FA weighting of connections and the Easy Lausanne scale 60 atlas (110 ROIs) (13) with connectomes undergoing consensus based thresholding at 75% and 50%. These values were chosen as they have been commonly used in structural connectomics (4, 19, 20).

Construction of Structural Connectivity Matrices

For structural connectivity matrices ROIs were defined as connected if a fibre originated in ROI 1 and terminated in ROI 2. Structural connections were weighted by streamline count and a cross-sectional area multiplier as implemented in SIFT2 (18). Probabilistic tractography as implemented in MRtrix3 creates a connectome composed of one upper triangle of a connectivity matrix. This is then copied to the lower triangle to generate a symmetric matrix of 74x74. As there is no consensus in the literature regarding the optimal graph thresholding strategy (21) and results can vary widely based on the chosen approach (22) SIFT2 was our preferred method of bias correction. Indeed the creators of SIFT2 argue against the use of matrix thresholding as it introduces an arbitrary threshold value (23). SIFT2 was chosen in preference to SIFT as it requires much less processing time and retains the full connectome. SIFT2 utilises information from the FOD to determine a cross sectional area for each streamline thereby generating streamline volume estimates between regions (18).

Currently in the literature there is no consensus regarding volume normalisation in connectome studies. There is a suggestion that volume normalisation may overcompensate

volume-driven effects on streamline count (24). In keeping with this in our previous study we analysed both volume normalised and un-normalised connectomes and showed that volume normalisation results in biologically implausible findings, which are likely spurious (4). In a subsequent study using the same data set presented here we performed two complimentary tractography approaches: connectomics and voxel connectivity profiles (VCPs) (10). Volume normalisation was performed in the VCP analyses as the tractography is performed at the voxel level. Results between the two approaches were consistent suggesting the limited amount of brain atrophy seen in preHD has a minimal effect on tractography. Previous work by our group has demonstrated low within-subject variability of diffusion metrics in manifest HD participants, suggesting atrophy does not cause significant distortion of the diffusion signal (25). Thus the more limited atrophy seen in preHD is unlikely to introduce systematic differences in connectome construction.

Regional White Matter Atrophy

For each cortical brain region connection strength was defined as either the sum of cortico-striatal connection weights, sum of connection weights from regions in the opposite hemisphere (inter-hemispheric) or sum of connection weights from regions in the same hemisphere (intra-hemispheric). Rate of change in connection strength over 24 months was defined in the same way. PreHD were normalised relative to controls using a Z-score. These were then transformed to give positive atrophy and rate of atrophy measures, where higher scores represent greater connection atrophy. The atrophy score was used in the cross-sectional analysis, while the rate of atrophy score was used in the longitudinal analysis.

Cross-sectional Analysis

For the cross-sectional analysis a Z-score was calculated as follows:

$$Z_C(i) = \frac{\mu\big(C_h(i)\big) - C_k(i)}{\sigma(C_h(i))}.$$

where i is the regional connection strength, k is preHD, h is healthy controls, C is connection strength, μ is mean and σ is standard deviation. This was then transformed to produce atrophy measures between -1 and 1, were positive measures represent greatest atrophy, using the following equation:

$$Z_{C-T}(i) = \tanh(Z_C(i)).$$

This resulted in a transformed Z-score for each cortical region for each preHD participant cortico-striatal, inter-hemispheric and intra-hemispheric connections. An average was then calculated across the preHD group resulting in a single transformed Z-score for each cortical region.

Longitudinal Analysis

For each preHD participant and for each connection a least squares line was fitted over the regional connection strengths across time points and the rate of connection atrophy defined as the gradient of the least squares line. A Z-score was then calculated using the following equation:

$$Z_R(i) = \frac{\mu\big(R_h(i)\big) - R_k(i)}{\sigma(R_h(i))}.$$

where R is the rate of change of connection strength. This was then transformed to produce rate of atrophy measures between -1 and 1, using the following equation:

$$Z_{R-T}(i) = \tanh(Z_R(i)).$$

This resulted in a transformed Z-score of rate of regional atrophy for cortico-striatal, inter-hemispheric and intra-hemispheric connections for each preHD participant. An average was then calculated across the preHD group resulting in a single transformed Z-score for each cortical region.

Mapping Gene Expression Data to MRI Space

Gene expression microarray data was used from the Allen Human brain atlas (26). This atlas is based on data from 6 post-mortem human brains with no known neuropsychiatric or neuropathological history (H0351.2001, H0351.2002, H0351.1009, H0351.1012, H0351.1015, H0351.1016). Five donors were male and one was female with a mean age 42.5yrs. Three were Caucasian, two were African-American and one was Hispanic. This data is freely available to download from AIBS (http://human.brain-map.org/static/download).

Maybrain software (https://github.com/rittman/maybrain) was used to match centroids of MRI regions to the closest AIBS region. The nearest gene expression profile to the ROI coordinates was used as the expression profile for that ROI. Therefore for each ROI only one tissue sample was used from the AIBS atlas. The sample coverage for the AIBS atlas varied from 255-291 cortical samples for the 4 participants with data from one hemisphere. For the 2 participants with data from both hemispheres one had 412 samples and the other 528. Probes were excluded that did not match to gene symbols in the AIBS data resulting in 20,737 genes included in the analysis. Expression data was then averaged across all samples from all donors. Data were also averaged across both hemispheres as two donors had data for both hemispheres, while four only had data for the left hemisphere. The maximum standard deviation across subjects for each gene probe in each brain region ranged from 0.1 to 4.6 (see Supplemental File 8). To account for this variability the mean and range of expression values for each brain region were calculated and regions excluded if they had values greater than

two standard deviations from either the mean or range. This resulted in the exclusion of two brain regions (right pars orbitalis and right rostral middle frontal), leaving a total of 68 cortical ROIs included in the analysis. Expression data were then normalised by calculating the Z-score across the 68 FreeSurfer regions. Similar approaches as those outlined above have been used when matching AIBS data to MRI atlases in other studies (27-29). Genetic data from outlier regions is likely to be unreliable. While it is difficult to pin point the exact reason for outlier regions in these analyses it may be that outlier regions represent sub-optimal matching between the AIBS and MRI atlases.

To investigate how robust results were to different combinations of AIBS participants, we also performed cortico-striatal, inter-hemispheric, intra-hemispheric cross-sectional analyses using using a leave one out approach. Average gene expression was calculated for 5 participants leaving one participant out in turn. A leave one out approach has been used in a previous study investigating regional gene expression and functional connectivity using the Allen institute of Brain Science human transcriptome atlas (28). We also repeated the cross-sectional analyses using permutations of 3 out of 6 AIBS brain samples resulting in a total of 8 permutations.

Statistical Analysis

All statistical analysis was performed in MATLAB *v*8.3. Partial least squares regression was used to investigate the association between gene transcriptome of the healthy brain and WM connectivity loss in preHD both cross-sectionally and longitudinally. Code used to perform this analysis was adapted from Whitaker et al. (29). The original code is freely available (https://github.com/KirstieJane/NSPN_WhitakerVertes_PNAS2016).

Partial least squares regression is a multivariate technique used to identify associations between response and predictor variables. In our case the predictor variable was

a 20,737 gene x 68. ROI matrix, as outlined above. For the cortico-striatal analysis the MRI data response variable was a 4 x 68 matrix of left and right caudate and putamen WM connectivity loss (preHD relative to controls) to 68 cortical ROIs. This was performed for both white matter atrophy (cross-sectional) and rate of white matter atrophy (longitudinal). For the inter-hemispheric analysis the MRI response variable was a vector of 1 x 68, representing WM inter-hemispheric connectivity loss for each cortical ROI. Similarly for the intra-hemispheric analysis the MRI response variable was a vector of 1 x 68, representing WM intra-hemispheric connectivity loss for each cortical ROI. For a cortical region inter-hemispheric connectivity was calculated as the sum of streamline volumes between that region and regions in the opposite hemisphere. Similarly intra-hemispheric connectivity was defined as the sum of streamline volumes between that region and regions in the same hemisphere. Atrophy scores were then calculated as using Z-scores and the tanh transform as described above.

As the greatest amount of variance was explained by the first PLS component, genes were ranked based on their contribution to this component. The error in estimating the weight of each gene was assessed by boot strapping and the ratio of the weight of each gene to its bootstrap standard deviation was used to rank the genes in descending order based on their contribution of the first component.

Random permutations of the gene predictor variable were also investigated to ensure results were not due to chance. To do this the randperm function in MATLAB was used to randomly reorder the predictor variable both in terms of genes and ROIs. Cross-sectional analyses were then re-run using the resulting predictor variables.

Partial least squares regression (PLS) is well suited for high dimensional data as it combines Principle components analysis (for dimension reduction) with linear regression. It is also well suited in the case when the number of predictor variables far exceeds the number

of observations – exactly the scenario we are dealing with having 20,737 gene expression (predictor variables) and 68 brain region (observations). In comparison, other multivariate methods such as canonical variance analysis (CVA) or linear discriminant analysis (LDA) require around 4-8 times observations than the predictor variables. Boulesteix et al. (30) have previously shown the utility of this approach in high dimensional datasets for e.g. tumor classification from transcriptome data, identification of relevant genes, survival analysis and modeling of gene networks and transcription factor activities. There are several previous studies that used PLS for the large gene expression datasets from the Allen Institute of Brain Science (AIBS) mouse and human brain transcriptome atlases (28, 29, 31).

Gene Ontology Enrichment Analysis

We used the gene ontology enrichment analysis and visualisation tool (GOrilla) (http://cbl-gorilla.cs.technion.ac.il) (32) to identify GO terms that were significantly enriched in the target gene list, based on the first PLS component. GOrilla GO terms are updated weekly. The target gene list is defined by finding the optimal hypergeometric tail probability over all possible partitions induced by gene ranking (see (32) for further details). Significance of a GO term is determined based on the rank of genes associated with that GO term and a false discovery rate (FDR) correction for multiple comparisons. This was performed for the first PLS component for the cortico-striatal, inter-hemispheric and intra-hemispheric analysis both cross-sectionally and longitudinally. We also removed general GO terms by excluding those with greater than 1000 genes in their classification, in keeping with other studies in the literature (28, 29). This allowed us to focus on specific gene sets as opposed to GO terms encompassing thousands of genes covering a range of processes. The reduce and visualize gene ontology tool REViGO (33) (http://revigo.irb.hr) was then used to summarise significant GO terms by removing redundant terms.

Overlap Between Gene Profiles and Huntington's Disease Related Genes

To investigate similarities between gene profiles in each analysis we identified which genes overlap in the top ranked 7,000 genes (based on target gene lists from top GO terms) from the cross-sectional cortico-striatal analysis and the intra-hemispheric analysis. We also assessed the probability of this overlap occurring greater than chance using a hypergeometric distribution as implemented in https://github.com/brentp/bio-playground/blob/master/utils/list_overlap_p.py. Gene ontology enrichment analysis was also repeated with overlap genes removed to assess whether this affected the resulting GO terms.

To further assess the relationship between gene ontologies we investigated the overlap between genes in the top gene ontology terms across analyses: "modulation of chemical synaptic transmission" and "mRNA metabolic process". Finally we investigated the overlap between top gene ontology terms and HD related genes. Gene lists for HD related genes for both the striatum and cortex were obtained from (34).

Cortical Regional Enrichment

We used ROI weights from the PLS analysis to assess which cortical regions where enriched for genes in the first PLS component for the cortico-striatal, inter-hemispheric and intra-hemispheric analysis. ROI weights were plotted for each analysis using BrainNet Viewer (35).

Enrichment for Huntington's Disease Related Genes

We also investigated whether genes showing abnormal transcription in human and animal models of HD were enriched greater than chance in the first PLS components of the cortico-striatal, inter-hemispheric and intra-hemispheric analyses. Gene lists were obtained from (34). These included 515 genes in the striatum and 25 in the cortex.

Gene lists for the striatum include the 6-month allelic series striatum from Langfelder et al. (34) and the human caudate nucleus (CN) data sets by Durrenberger et al. (36) and Hodges et al. (37) are reported. Each striatal gene satisfies the following criteria: FDR<0.05 in the allelic series striatum, FDR<0.1 in each of the human data sets, and same sign of fold change across all 3 data sets. For the cortex the gene lists include the allelic series 6-month cortex, Brodmann area (BA) 4 and BA9 data by Hodges et al. (37), and prefrontal cortex (PFC) and visual cortex (VC) data from the Harvard Brain Tissue Resource Centre are reported (38). Each cortical gene satisfies the following criteria: FDR<0.05 in the allelic series cortex, FDR<0.1 in at least 3 of the 4 of the human data sets, and same sign of fold change in the allelic series cortex and at least 3 of the 4 human data sets. Genes in the Langfelder lists not included in the AIBS gene set were excluded; this resulted in the exclusion of 28 striatum genes.

The mean PLS weight of candidate gene sets were compared against the mean PLS weight of 1000 random permutations of genes. A p-value was calculated based on the number of times in 1000 that the random gene list showed a higher mean rank than the candidate gene list. We also investigated whether HD related genes were more strongly enriched in these gene lists than other biologically plausible gene sets, chosen at random. In order to do this gene sets from known gene ontologies were downloaded from the molecular signatures database (MSigBD) (http://software.broadinstitute.org/gsea/msigdb/). A p-value was calculated based on the number of times that the MSigBD gene list showed a higher mean rank than the candidate gene list. This was performed for the 515 striatum HD genes and MSigBD gene lists truncated at 515 (306 lists in total). In order to investigate smaller alternative gene sets the top 25 striatum HD genes were also compared with MSigBD gene lists truncated at 25 (3,633 lists in total).

To further investigate the relationship between changes in gene expression in HD relative to controls and cortico-striatal WM loss we performed correlations between the log2 fold change in the Hodges (37), Durrenberger (36) and Langfelder studies (34) for the 515 striatum gene set and the PLS weights from the cross-sectional cortico-striatal analysis.

Enrichment for Alternative Gene Sets

Enrichment of the PLS components of the cortico-striatal, inter-hemispheric and intra-hemispheric analyses were also tested for a range of other gene sets. We included a set of human supragranular genes (n = 19) as these have been implicated in long-range connectivity (39) and we have previously shown cortico-striatal connections to have the longest topological length of the white connections subtypes investigated here (10). Genes specific to oligodendroctyes (n = 94) (40) were also included to investigate whether white matter loss may be driven by axonal or myelination dysfunction. Finally, genes involved in cell cycle metabolism (n = 252) (http://www.bmrb.wisc.edu/data_library/Genes/Metabolic_Pathways/Cell_cycle.html) were included as mutant huntingtin has been shown to cause cell cycle abnormalities (41).

Supplemental Figures

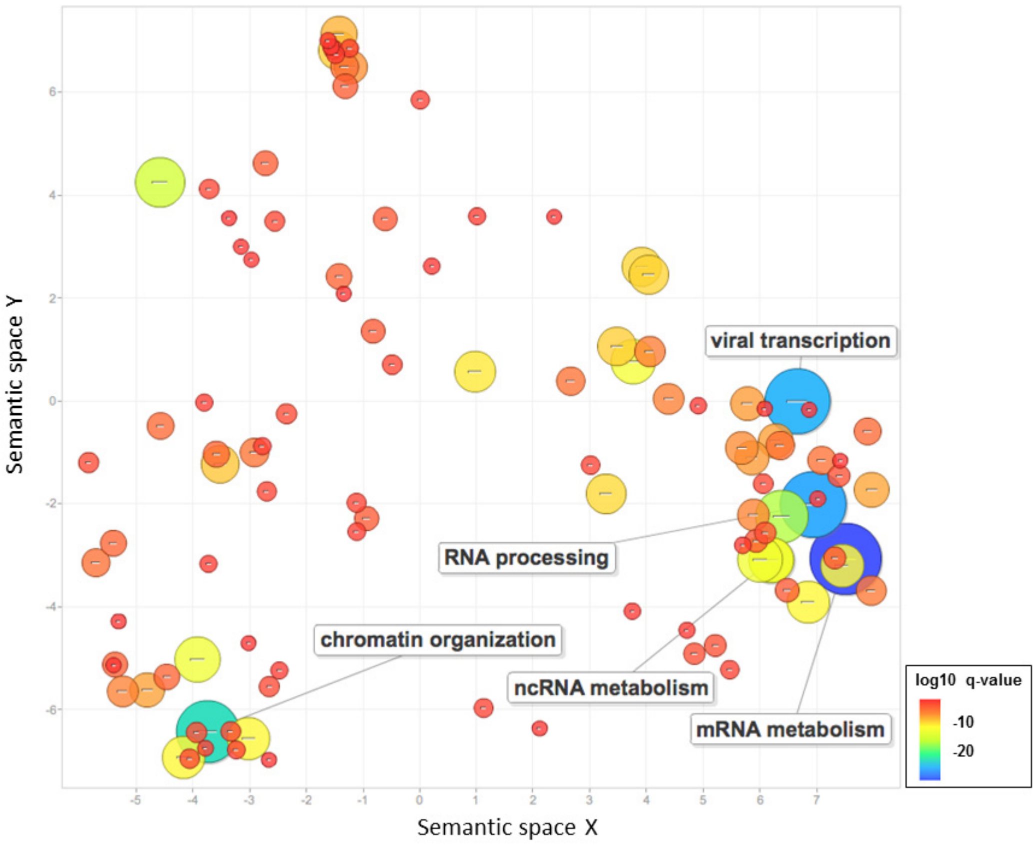

Figure S1. Cortico-striatal longitudinal analysis semantic similarity scatter plot: Significant gene ontology (GO) terms for biological processes associated with the first component of the partial least squares (PLS) analysis are plotted in semantic space, where similar terms are clustered together. The top 5 most significant GO terms are labelled for each analysis. Redundant GO terms and those associated with greater than 1000 genes have been excluded. Markers are scaled based on the log10 q-value for the significance of each GO term. Large blue circles are highly significant, while red circles are less significant (see colour bar).

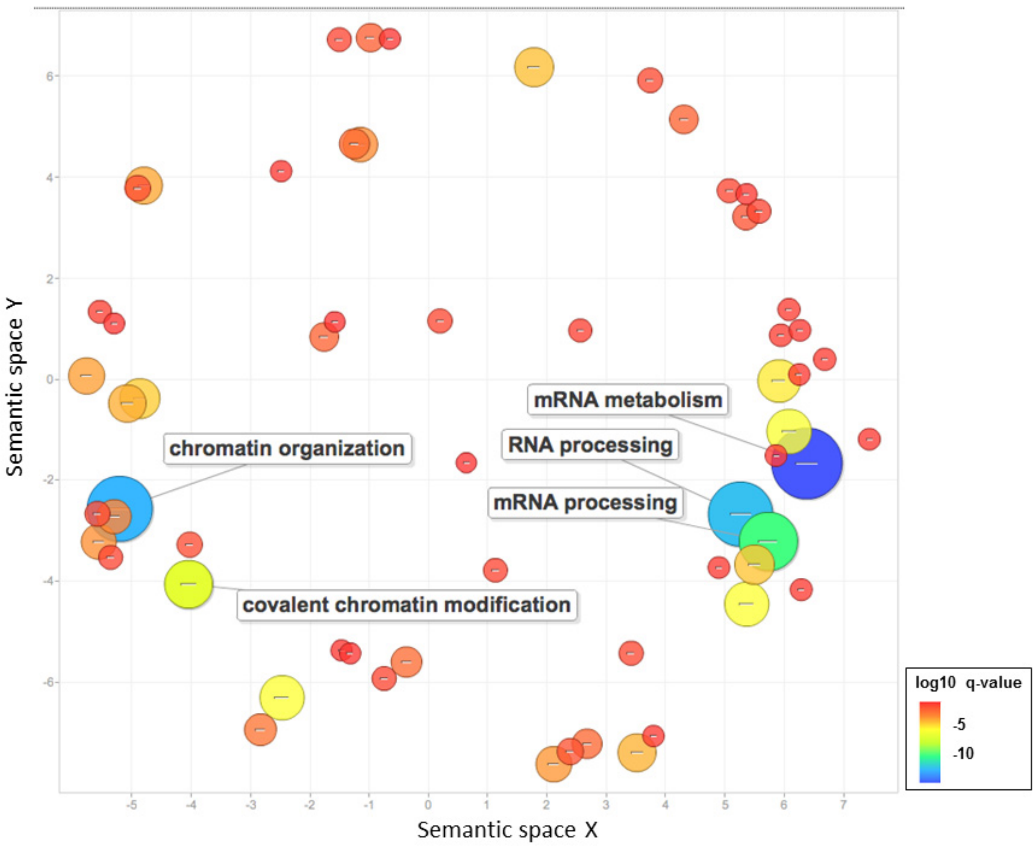

Figure S2. Inter-hemispheric longitudinal analysis semantic similarity scatter plot: Significant gene ontology (GO) terms for biological processes associated with the first component of the partial least squares (PLS) analysis are plotted in semantic space, where similar terms are clustered together. The top 5 most significant GO terms are labelled for each analysis. Redundant GO terms and those associated with greater than 1000 genes have been excluded. Markers are scaled based on the log10 q-value for the significance of each GO term. Large blue circles are highly significant, while red circles are less significant (see colour bar).

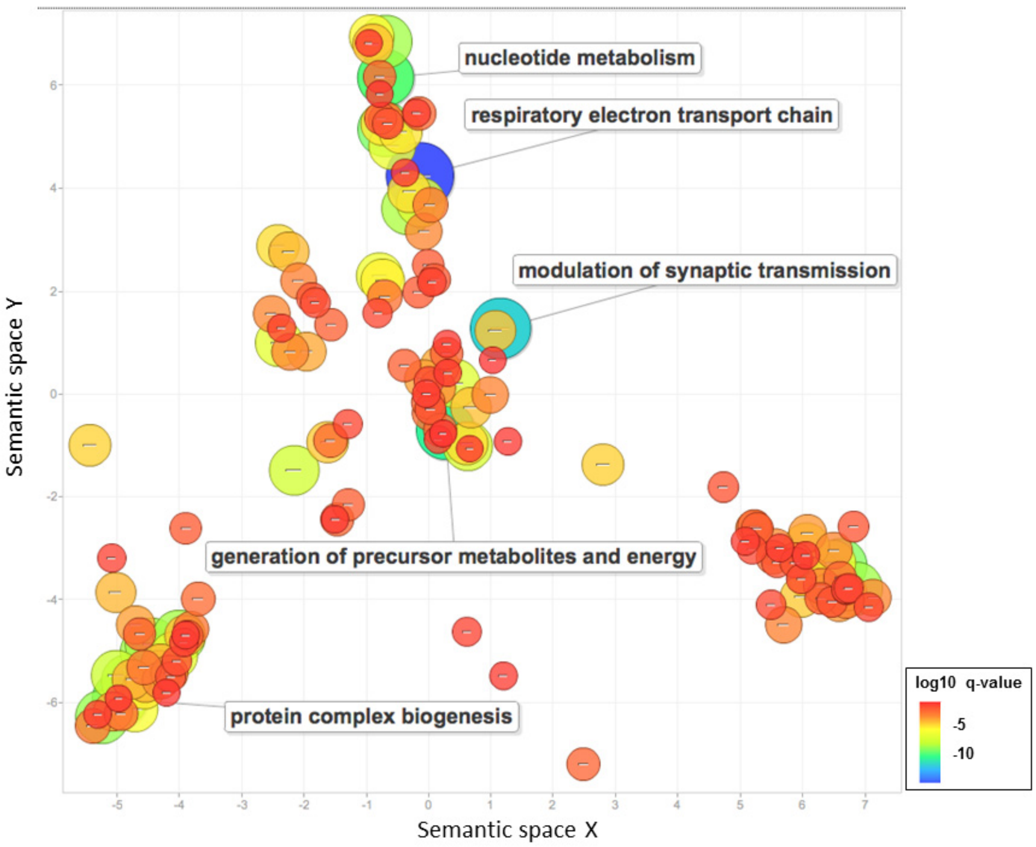

Figure S3. Intra-hemispheric longitudinal analysis semantic similarity scatter plot: Significant gene ontology (GO) terms for biological processes associated with the first component of the partial least squares (PLS) analysis are plotted in semantic space, where similar terms are clustered together. The top 5 most significant GO terms are labelled for each analysis. Redundant GO terms and those associated with greater than 1000 genes have been excluded. Markers are scaled based on the log10 q-value for the significance of each GO term. Large blue circles are highly significant, while red circles are less significant (see colour bar).

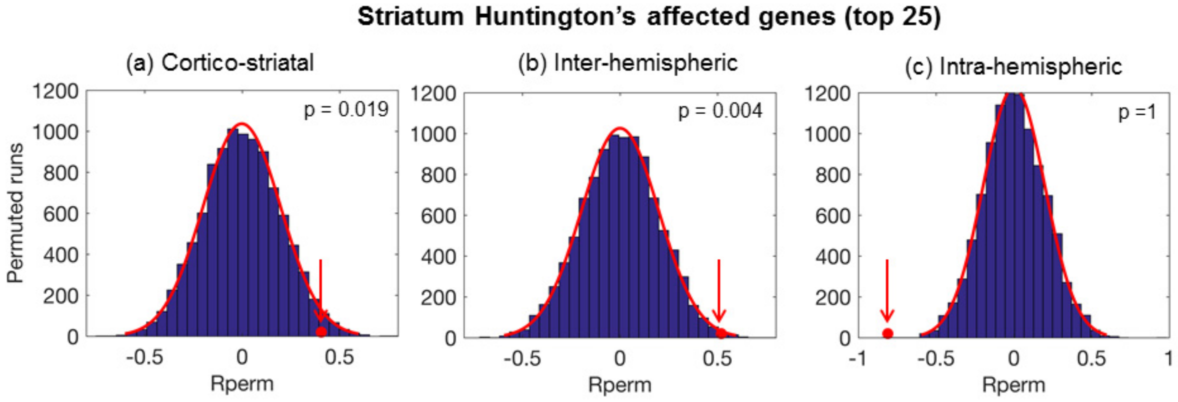

Figure S4. Enrichment of top 25 striatum genes showing abnormal transcription in Huntington's disease (as defined by lowest Hodges q-value) in the first PLS components of cortico-striatal cross-sectional analyses. Red circle illustrates the mean weight (on the x-axis) for the gene list of interest in the first PLS component. The y-axis represents the number of permutations of random genes from the first PLS component. Gene lists over expressed in the first PLS component have a mean great than that of the random permutations (red circle to the right of the permutation distribution).

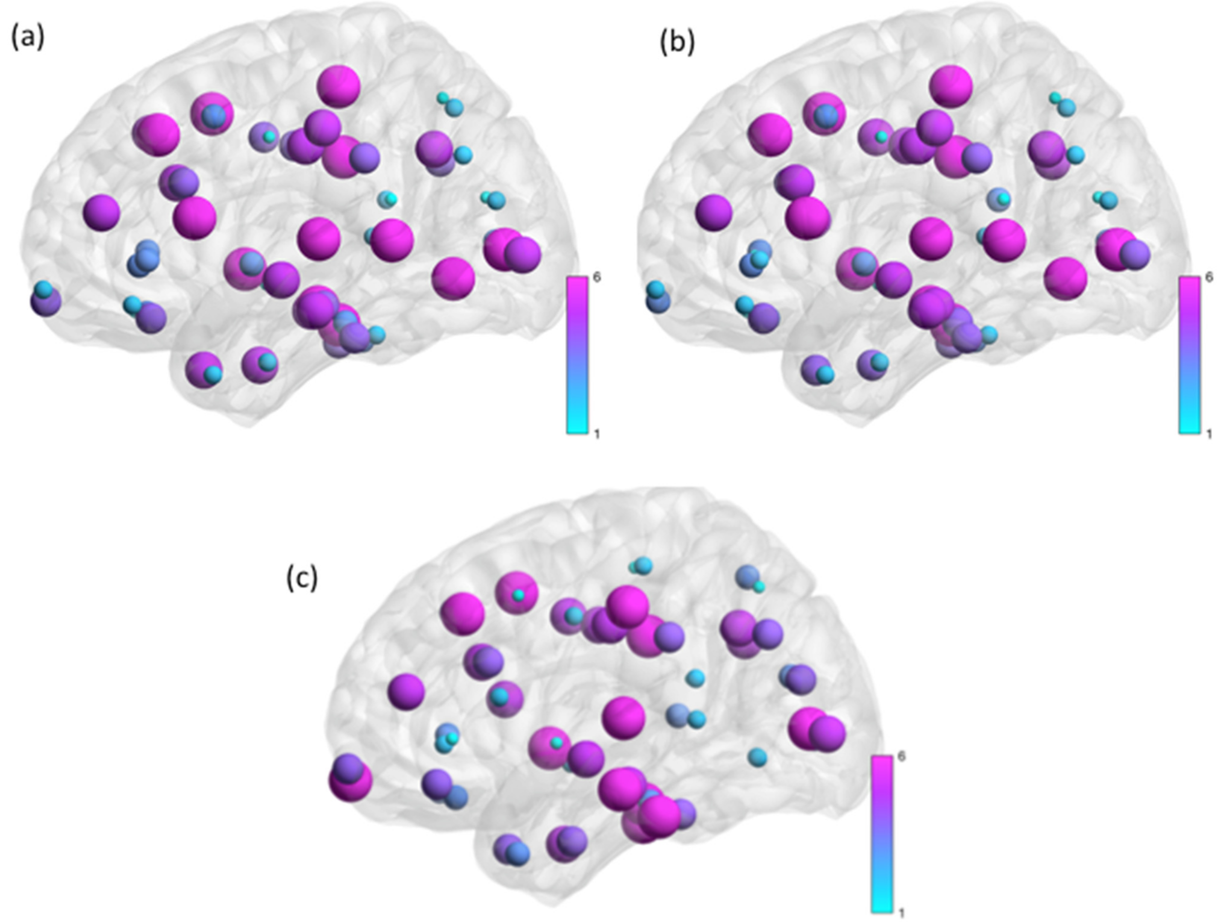

Figure S5. Random permutation ROI weights for cross-sectional partial least squares regression analyses. (a) Cortico-striatal (b) Inter-hemispheric (c) Intra-hemispheric. Brain regions displayed on brain mesh. Size and colour of region indicates size of ROI weight (ranked from smallest-largest, 1-6). See colour map.

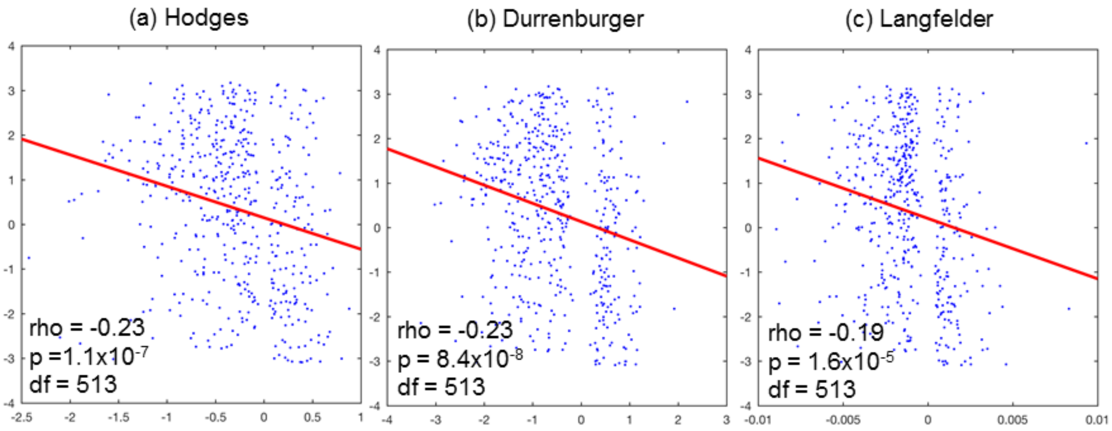

Figure S6. Correlation between PLS1 cortico-striatal weights and log2 fold change in human HD (Hodges and Durrenberger) and animal HD model (Langfelder) studies. The red line represents a least squares regression line, rho = correlation coefficient, p = p-value and df = degrees of freedom.

Table S1. Cortico-striatal, inter-hemispheric and intra-hemispheric longitudinal analysis: Gene ontology (GO) terms for biological processes associated with top ranking genes from the first component of the partial least squares (PLS) analysis. The top 5 most significant GO terms are displayed for each analysis. Full tables can be found in supplementary file 2. Redundant GO terms and those associated with greater than 1000 genes have been excluded. B – total number of genes associated with a specific GO term, n – number of genes in target set, b – is the number of genes in the intersection. Enrichment (E) = (b/n) / (B/total number of genes). See (32) for further details.

**PLS1 Cortico-striatal Longitudinal**							
**GO Term**	**Description**	**P-value**	**FDR q-value**	**Enrichment**	**B**	**n**	**b**
GO:0016071	mRNA metabolic process	3.51E-33	1.35E-30	1.81	593	5324	322
GO:0006396	RNA processing	7.90E-29	2.77E-26	1.64	806	5324	397
GO:0019083	viral transcription	3.28E-28	1.12E-25	2.86	99	5251	84
GO:0006325	chromatin organization	9.85E-26	3.22E-23	1.77	657	4520	296
GO:0000184	nuclear-transcribed mRNA catabolic process, nonsense-mediated decay	2.16E-19	6.78E-17	2.53	102	5298	77
**PLS1 Inter-hemispheric Longitudinal**							
**GO Term**	**Description**	**P-value**	**FDR q-value**	**Enrichment**	**B**	**n**	**b**
GO:0016071	mRNA metabolic process	3.98E-16	1.67E-13	1.48	593	6539	323
GO:0006325	chromatin organization	4.09E-14	1.47E-11	1.4	657	6476	337
GO:0006396	RNA processing	7.78E-14	2.66E-11	1.36	806	6410	397
GO:0006397	mRNA processing	6.16E-12	2.02E-09	1.49	402	6323	213
GO:0016569	covalent chromatin modification	4.62E-09	1.36E-06	1.38	455	6476	230
**PLS1 Intra-hemispheric Longitudinal**							
**GO Term**	**Description**	**P-value**	**FDR q-value**	**Enrichment**	**B**	**n**	**b**
GO:0022904	respiratory electron transport chain	2.29E-13	1.15E-09	2.71	92	3917	55
GO:0050804	modulation of chemical synaptic transmission	2.95E-11	6.35E-08	1.84	297	3658	113
GO:0006091	generation of precursor metabolites and energy	1.42E-10	2.38E-07	1.64	263	5414	132
GO:0009117	nucleotide metabolic process	4.97E-10	7.48E-07	1.88	418	2364	105
GO:0070271	protein complex biogenesis	9.42E-10	1.01E-06	2.46	81	4186	47

Table S2. ROI weights from first PLS components. BG – basal ganglia, IH – Inter-hemispheric, IA – Intra-hemispheric, cross – cross-sectional, long – longitudinal**.** Weights ordered for basal ganglia cross-sectional analysis, decreasing strongest to weakest.

Region	CS cross	IH cross	IA cross	CS long	IH long	IA long
R.inferiorparietal	0.22034	0.028159	-0.077541	-0.11582	-0.068989	0.15782
R.precentral	0.20856	0.047965	-0.088025	-0.12867	-0.059647	0.17176
R.superiorparietal	0.20856	0.047965	-0.088025	-0.12867	-0.059647	0.17176
L.cuneus	0.15725	0.10562	-0.12229	-0.12238	-0.10801	0.13927
L.inferiorparietal	0.15725	0.10562	-0.12229	-0.12238	-0.10801	0.13927
L.isthmuscingulate	0.15725	0.10562	-0.12229	-0.12238	-0.10801	0.13927
L.lateraloccipital	0.15725	0.10562	-0.12229	-0.12238	-0.10801	0.13927
L.paracentral	0.15725	0.10562	-0.12229	-0.12238	-0.10801	0.13927
L.pericalcarine	0.15725	0.10562	-0.12229	-0.12238	-0.10801	0.13927
L.posteriorcingulate	0.15725	0.10562	-0.12229	-0.12238	-0.10801	0.13927
L.precuneus	0.15725	0.10562	-0.12229	-0.12238	-0.10801	0.13927
L.superiorparietal	0.15725	0.10562	-0.12229	-0.12238	-0.10801	0.13927
L.supramarginal	0.15725	0.10562	-0.12229	-0.12238	-0.10801	0.13927
R.isthmuscingulate	0.15725	0.10562	-0.12229	-0.12238	-0.10801	0.13927
R.paracentral	0.15725	0.10562	-0.12229	-0.12238	-0.10801	0.13927
R.posteriorcingulate	0.15725	0.10562	-0.12229	-0.12238	-0.10801	0.13927
R.precuneus	0.15725	0.10562	-0.12229	-0.12238	-0.10801	0.13927
R.supramarginal	0.15725	0.10562	-0.12229	-0.12238	-0.10801	0.13927
R.caudalmiddlefrontal	0.14542	0.10192	-0.093706	-0.090327	-0.16404	0.11031
R.postcentral	0.14542	0.10192	-0.093706	-0.090327	-0.16404	0.11031
R.cuneus	0.12658	0.097045	-0.10093	-0.1211	-0.078746	0.10593
L.postcentral	0.11425	0.099365	-0.10889	-0.12017	-0.094212	0.1137
L.caudalmiddlefrontal	0.054748	0.11976	-0.11586	-0.10097	-0.12061	0.097573
L.transversetemporal	0.030688	0.11459	-0.10071	-0.10298	-0.055668	0.067514
R.caudalanteriorcingulate	0.030688	0.11459	-0.10071	-0.10298	-0.055668	0.067514
L.precentral	0.0041809	0.1269	-0.10137	-0.089321	-0.092781	0.062488
R.superiorfrontal	0.0041809	0.1269	-0.10137	-0.089321	-0.092781	0.062488
L.caudalanteriorcingulate	0.00108	0.11641	-0.10077	-0.099388	-0.054861	0.055352
L.parsopercularis	0.00108	0.11641	-0.10077	-0.099388	-0.054861	0.055352
L.superiortemporal	0.00108	0.11641	-0.10077	-0.099388	-0.054861	0.055352
L.insula	0.00108	0.11641	-0.10077	-0.099388	-0.054861	0.055352
L.parsorbitalis	-0.013671	0.13099	-0.10188	-0.083891	-0.11266	0.051551
L.parstriangularis	-0.013671	0.13099	-0.10188	-0.083891	-0.11266	0.051551
L.rostralmiddlefrontal	-0.026883	0.1453	-0.118	-0.095567	-0.12	0.061948
L.superiorfrontal	-0.026883	0.1453	-0.118	-0.095567	-0.12	0.061948
L.frontalpole	-0.026883	0.1453	-0.118	-0.095567	-0.12	0.061948
R.frontalpole	-0.026883	0.1453	-0.118	-0.095567	-0.12	0.061948
L.entorhinal	-0.077019	-0.16519	0.12322	0.10631	0.20821	-0.10852
L.medialorbitofrontal	-0.077019	-0.16519	0.12322	0.10631	0.20821	-0.10852
L.temporalpole	-0.077019	-0.16519	0.12322	0.10631	0.20821	-0.10852
R.entorhinal	-0.077019	-0.16519	0.12322	0.10631	0.20821	-0.10852

Region	CS cross	IH cross	IA cross	CS long	IH long	IA long
R.lateralorbitofrontal	-0.077019	-0.16519	0.12322	0.10631	0.20821	-0.10852
R.medialorbitofrontal	-0.077019	-0.16519	0.12322	0.10631	0.20821	-0.10852
R.temporalpole	-0.077019	-0.16519	0.12322	0.10631	0.20821	-0.10852
L.fusiform	-0.11678	-0.11347	0.12528	0.14275	0.07509	-0.13103
L.inferiortemporal	-0.11678	-0.11347	0.12528	0.14275	0.07509	-0.13103
L.lateralorbitofrontal	-0.11678	-0.11347	0.12528	0.14275	0.07509	-0.13103
L.lingual	-0.11678	-0.11347	0.12528	0.14275	0.07509	-0.13103
L.middletemporal	-0.11678	-0.11347	0.12528	0.14275	0.07509	-0.13103
L.parahippocampal	-0.11678	-0.11347	0.12528	0.14275	0.07509	-0.13103
L.rostralanteriorcingulate	-0.11678	-0.11347	0.12528	0.14275	0.07509	-0.13103
L.hippocampus	-0.11678	-0.11347	0.12528	0.14275	0.07509	-0.13103
R.hippocampus	-0.11678	-0.11347	0.12528	0.14275	0.07509	-0.13103
R.parahippocampal	-0.11678	-0.11347	0.12528	0.14275	0.07509	-0.13103
R.rostralanteriorcingulate	-0.11678	-0.11347	0.12528	0.14275	0.07509	-0.13103
R.fusiform	-0.12353	-0.094844	0.14257	0.16319	-0.0012139	-0.15301
R.lateraloccipital	-0.12353	-0.094844	0.14257	0.16319	-0.0012139	-0.15301
R.lingual	-0.12353	-0.094844	0.14257	0.16319	-0.0012139	-0.15301
R.pericalcarine	-0.12353	-0.094844	0.14257	0.16319	-0.0012139	-0.15301
R.transversetemporal	-0.12353	-0.094844	0.14257	0.16319	-0.0012139	-0.15301
L.bankssts	-0.12417	-0.11555	0.12622	0.12119	0.089642	-0.11748
R.parstriangularis	-0.12699	-0.1361	0.096136	0.094345	0.14245	-0.058084
R.bankssts	-0.13821	-0.14829	0.15138	0.11965	0.19037	-0.13649
R.inferiortemporal	-0.13821	-0.14829	0.15138	0.11965	0.19037	-0.13649
R.middletemporal	-0.13821	-0.14829	0.15138	0.11965	0.19037	-0.13649
R.parsopercularis	-0.13821	-0.14829	0.15138	0.11965	0.19037	-0.13649
R.superiortemporal	-0.13821	-0.14829	0.15138	0.11965	0.19037	-0.13649
R.insula	-0.13821	-0.14829	0.15138	0.11965	0.19037	-0.13649

Supplemental References

1. Tabrizi SJ, Langbehn DR, Leavitt BR, Roos RA, Durr A, Craufurd D, et al. (2009): Biological and clinical manifestations of Huntington's disease in the longitudinal TRACK-HD study: cross-sectional analysis of baseline data. *Lancet Neurol*. 8:791-801.
2. Penney JB, Jr., Vonsattel JP, MacDonald ME, Gusella JF, Myers RH (1997): CAG repeat number governs the development rate of pathology in Huntington's disease. *Ann Neurol*. 41:689-692.
3. Desikan RS, Segonne F, Fischl B, Quinn BT, Dickerson BC, Blacker D, et al. (2006): An automated labeling system for subdividing the human cerebral cortex on MRI scans into gyral based regions of interest. *Neuroimage*. 31:968-980.
4. McColgan P, Seunarine KK, Razi A, Cole JH, Gregory S, Durr A, et al. (2015): Selective vulnerability of Rich Club brain regions is an organizational principle of structural connectivity loss in Huntington's disease. *Brain*. 138:3327-3344.
5. Tabrizi SJ, Scahill RI, Durr A, Roos RA, Leavitt BR, Jones R, et al. (2011): Biological and clinical changes in premanifest and early stage Huntington's disease in the TRACK-HD study: the 12-month longitudinal analysis. *Lancet Neurol*. 10:31-42.
6. Tabrizi SJ, Reilmann R, Roos RA, Durr A, Leavitt B, Owen G, et al. (2012): Potential endpoints for clinical trials in premanifest and early Huntington's disease in the TRACK-HD study: analysis of 24 month observational data. *Lancet Neurol*. 11:42-53.
7. Faria AV, Ratnanather JT, Tward DJ, Lee DS, van den Noort F, Wu D, et al. (2016): Linking white matter and deep gray matter alterations in premanifest Huntington disease. *Neuroimage Clin*. 11:450-460.
8. van den Bogaard SJ, Dumas EM, Acharya TP, Johnson H, Langbehn DR, Scahill RI, et al. (2011): Early atrophy of pallidum and accumbens nucleus in Huntington's disease. *J Neurol*. 258:412-420.
9. Hibar DP, Stein JL, Renteria ME, Arias-Vasquez A, Desrivieres S, Jahanshad N, et al. (2015): Common genetic variants influence human subcortical brain structures. *Nature*. 520:224-229.
10. McColgan P, Seunarine KK, Gregory S, Razi A, Papoutsi M, Long JD, et al. (2017): Topological length of white matter connections predicts their rate of atrophy in premanifest Huntington's disease. *JCI Insight*. 2.
11. McColgan P, Razi A, Gregory S, Seunarine KK, Durr A, R ACR, et al. (2017): Structural and functional brain network correlates of depressive symptoms in premanifest Huntington's disease. *Hum Brain Mapp*. 38:2819-2829.
12. McColgan P, Gregory S, Razi A, Seunarine KK, Gargouri F, Durr A, et al. (2017): White matter predicts functional connectivity in premanifest Huntington's disease. *Ann Clin Transl Neurol*. 4:106-118.
13. Cammoun L, Gigandet X, Meskaldji D, Thiran JP, Sporns O, Do KQ, et al. (2012): Mapping the human connectome at multiple scales with diffusion spectrum MRI. *J Neurosci Methods*. 203:386-397.
14. Tzourio-Mazoyer N, Landeau B, Papathanassiou D, Crivello F, Etard O, Delcroix N, et al. (2002): Automated anatomical labeling of activations in SPM using a macroscopic anatomical parcellation of the MNI MRI single-subject brain. *Neuroimage*. 15:273-289.

15. Smith SM (2002): Fast robust automated brain extraction. *Hum Brain Mapp*. 17:143-155.
16. Tournier JD, Calamante F, Connelly A (2012): MRtrix: Diffusion tractography in crossing fiber regions. *Imaging Systems and Technology*. 22:53-56.
17. Modat M, Ridgway GR, Taylor ZA, Lehmann M, Barnes J, Hawkes DJ, et al. (2010): Fast free-form deformation using graphics processing units. *Comput Methods Programs Biomed*. 98:278-284.
18. Smith RE, Tournier JD, Calamante F, Connelly A (2015): SIFT2: Enabling dense quantitative assessment of brain white matter connectivity using streamlines tractography. *Neuroimage*. 119:338-351.
19. van den Heuvel MP, Sporns O (2011): Rich-club organization of the human connectome. *J Neurosci*. 31:15775-15786.
20. van den Heuvel MP, Kahn RS, Goni J, Sporns O (2012): High-cost, high-capacity backbone for global brain communication. *Proc Natl Acad Sci U S A*. 109:11372-11377.
21. Qi S, Meesters S, Nicolay K, Romeny BM, Ossenblok P (2015): The influence of construction methodology on structural brain network measures: A review. *J Neurosci Methods*. 253:170-182.
22. Garrison KA, Scheinost D, Finn ES, Shen X, Constable RT (2015): The (in)stability of functional brain network measures across thresholds. *Neuroimage*.
23. Yeh CH, Smith RE, Liang X, Calamante F, Connelly A (2016): Correction for diffusion MRI fibre tracking biases: The consequences for structural connectomic metrics. *Neuroimage*.
24. Zalesky A, Fornito A (2009): A DTI-derived measure of cortico-cortical connectivity. *IEEE Trans Med Imaging*. 28:1023-1036.
25. Cole JH, Farmer RE, Rees EM, Johnson HJ, Frost C, Scahill RI, et al. (2014): Test-Retest Reliability of Diffusion Tensor Imaging in Huntington's Disease. *PLoS Curr*. 6.
26. Hawrylycz M, Miller JA, Menon V, Feng D, Dolbeare T, Guillozet-Bongaarts AL, et al. (2015): Canonical genetic signatures of the adult human brain. *Nat Neurosci*. 18:1832-1844.
27. Rittman T, Rubinov M, Vertes PE, Patel AX, Ginestet CE, Ghosh BC, et al. (2016): Regional expression of the MAPT gene is associated with loss of hubs in brain networks and cognitive impairment in Parkinson disease and progressive supranuclear palsy. *Neurobiol Aging*. 48:153-160.
28. Vertes PE, Rittman T, Whitaker KJ, Romero-Garcia R, Vasa F, Kitzbichler MG, et al. (2016): Gene transcription profiles associated with inter-modular hubs and connection distance in human functional magnetic resonance imaging networks. *Philos Trans R Soc Lond B Biol Sci*. 371.
29. Whitaker KJ, Vertes PE, Romero-Garcia R, Vasa F, Moutoussis M, Prabhu G, et al. (2016): Adolescence is associated with genomically patterned consolidation of the hubs of the human brain connectome. *Proc Natl Acad Sci U S A*. 113:9105-9110.
30. Boulesteix AL, Strimmer K (2007): Partial least squares: a versatile tool for the analysis of high-dimensional genomic data. *Brief Bioinform*. 8:32-44.

31. Rubinov M, Ypma RJ, Watson C, Bullmore ET (2015): Wiring cost and topological participation of the mouse brain connectome. *Proc Natl Acad Sci U S A*. 112:10032-10037.

32. Eden E, Navon R, Steinfeld I, Lipson D, Yakhini Z (2009): GOrilla: a tool for discovery and visualization of enriched GO terms in ranked gene lists. *BMC Bioinformatics*. 10:48.

33. Supek F, Bosnjak M, Skunca N, Smuc T (2011): REVIGO summarizes and visualizes long lists of gene ontology terms. *PLoS One*. 6:e21800.

34. Langfelder P, Cantle JP, Chatzopoulou D, Wang N, Gao F, Al-Ramahi I, et al. (2016): Integrated genomics and proteomics define huntingtin CAG length-dependent networks in mice. *Nat Neurosci*. 19:623-633.

35. Xia M, Wang J, He Y (2013): BrainNet Viewer: a network visualization tool for human brain connectomics. *PLoS One*. 8:e68910.

36. Durrenberger PF, Fernando FS, Kashefi SN, Bonnert TP, Seilhean D, Nait-Oumesmar B, et al. (2015): Common mechanisms in neurodegeneration and neuroinflammation: a BrainNet Europe gene expression microarray study. *J Neural Transm (Vienna)*. 122:1055-1068.

37. Hodges A, Strand AD, Aragaki AK, Kuhn A, Sengstag T, Hughes G, et al. (2006): Regional and cellular gene expression changes in human Huntington's disease brain. *Hum Mol Genet*. 15:965-977.

38. Zhang B, Gaiteri C, Bodea LG, Wang Z, McElwee J, Podtelezhnikov AA, et al. (2013): Integrated systems approach identifies genetic nodes and networks in late-onset Alzheimer's disease. *Cell*. 153:707-720.

39. Krienen FM, Yeo BT, Ge T, Buckner RL, Sherwood CC (2016): Transcriptional profiles of supragranular-enriched genes associate with corticocortical network architecture in the human brain. *Proc Natl Acad Sci U S A*. 113:E469-478.

40. Cahoy JD, Emery B, Kaushal A, Foo LC, Zamanian JL, Christopherson KS, et al. (2008): A transcriptome database for astrocytes, neurons, and oligodendrocytes: a new resource for understanding brain development and function. *J Neurosci*. 28:264-278.

41. Molina-Calavita M, Barnat M, Elias S, Aparicio E, Piel M, Humbert S (2014): Mutant huntingtin affects cortical progenitor cell division and development of the mouse neocortex. *J Neurosci*. 34:10034-10040.
